# MLK4 regulates DNA damage response and promotes triple-negative breast cancer chemoresistance

**DOI:** 10.1038/s41419-021-04405-0

**Published:** 2021-11-27

**Authors:** Dawid Mehlich, Michał Łomiak, Aleksandra Sobiborowicz, Alicja Mazan, Dagmara Dymerska, Łukasz M. Szewczyk, Anna Mehlich, Agnieszka Borowiec, Monika K. Prełowska, Adam Gorczyński, Paweł Jabłoński, Ewa Iżycka-Świeszewska, Dominika Nowis, Anna A. Marusiak

**Affiliations:** 1 Laboratory of Molecular OncoSignalling, IMol Polish Academy of Sciences, Warsaw, Poland; 2 Doctoral School of Medical University of Warsaw, Warsaw, Poland; 3 Laboratory of Experimental Medicine, Medical University of Warsaw, Warsaw, Poland; 4 Centre of New Technologies, University of Warsaw, Warsaw, Poland; 5 ReMedy International Research Agenda Unit, IMol Polish Academy of Sciences, Warsaw, Poland; 6 Laboratory of Molecular Neurobiology, Centre of New Technologies, University of Warsaw, Warsaw, Poland; 7 Department of Internal Diseases Endocrinology and Diabetes, Medical University of Warsaw, Warsaw, Poland; 8 Department of Pathology and Neuropathology, Faculty of Health Sciences, Medical University of Gdansk, Gdansk, Poland; 9 Department of Pathomorphology, Copernicus P.L., Gdansk, Poland; 10 Department of Immunology, Medical University of Warsaw, Warsaw, Poland; 11 Present Address: Department of Experimental and Clinical Physiology, Medical University of Warsaw, Warsaw, Poland; 12 Present Address: Department of Oncology, Microbiology and Immunology, Faculty of Science and Medicine, University of Fribourg, Fribourg, Switzerland

**Keywords:** Chemotherapy, Oncogenes, Cell signalling, Breast cancer

## Abstract

Chemoresistance constitutes a major challenge in the treatment of triple-negative breast cancer (TNBC). Mixed-Lineage Kinase 4 (MLK4) is frequently amplified or overexpressed in TNBC where it facilitates the aggressive growth and migratory potential of breast cancer cells. However, the functional role of MLK4 in resistance to chemotherapy has not been investigated so far. Here, we demonstrate that MLK4 promotes TNBC chemoresistance by regulating the pro-survival response to DNA-damaging therapies. We observed that MLK4 knock-down or inhibition sensitized TNBC cell lines to chemotherapeutic agents in vitro. Similarly, MLK4-deficient cells displayed enhanced sensitivity towards doxorubicin treatment in vivo. MLK4 silencing induced persistent DNA damage accumulation and apoptosis in TNBC cells upon treatment with chemotherapeutics. Using phosphoproteomic profiling and reporter assays, we demonstrated that loss of MLK4 reduced phosphorylation of key DNA damage response factors, including ATM and CHK2, and compromised DNA repair via non-homologous end-joining pathway. Moreover, our mRNA-seq analysis revealed that MLK4 is required for DNA damage-induced expression of several NF-кB-associated cytokines, which facilitate TNBC cells survival. Lastly, we found that high MLK4 expression is associated with worse overall survival of TNBC patients receiving anthracycline-based neoadjuvant chemotherapy. Collectively, these results identify a novel function of MLK4 in the regulation of DNA damage response signaling and indicate that inhibition of this kinase could be an effective strategy to overcome TNBC chemoresistance.

## Introduction

Triple-negative breast cancer (TNBC) is a subtype of breast cancer characterized by the absence of estrogen and progesterone receptors and the lack of HER2 amplification or overexpression. It accounts for 15–20% of all invasive breast cancers and is associated with an inferior prognosis compared with other breast cancer subtypes [1]. In the absence of known druggable molecular drivers, anthracyclines and taxanes-based chemotherapy is the mainstay of systemic treatment for TNBC [2]. Approximately 30% of women with TNBC who receive preoperative neoadjuvant chemotherapy (NAC) achieve a pathological complete response (pCR). Achieving pCR at the time of surgery has been correlated with a favorable prognosis in clinical trials [3, 4]. Nevertheless, both intrinsic and acquired resistance to chemotherapy leads to high rates of relapse and poor outcomes in most patients. Therefore, there is a need to identify novel molecular targets that could be exploited to overcome TNBC chemoresistance.

Accumulating evidence suggests that the development of resistance to genotoxic chemotherapy can be caused by aberrant regulation and overexpression of different components of the DNA repair pathways in cancer cells [5]. Ataxia telangiectasia mutated (ATM) kinase is one of the central kinases involved in the cellular response to DNA double strand breaks (DSBs) induced by chemotherapy. Following induction of DSBs, ATM is activated through auto- or trans- phosphorylation at Ser1981 and phosphorylates numerous targets to initiate DNA damage response (DDR), which may involve cell cycle arrest, repair of DNA lesions, and transcriptional reprogramming [6–8]. Many reports linked increased ATM and DDR signaling with the survival of cancer cells after chemotherapy, rendering this pathway an attractive target for overcoming cancer chemoresistance [9–14]. However, despite recent advancements in our understanding of ATM signaling, the complex mechanisms involved in its activation and functional role in resistance to chemotherapy are not yet fully resolved.

Mixed-Lineage Kinase 4 (MLK4) is a member of the Mixed-Lineage Kinases (MLKs) family of serine/threonine kinases. Large-scale genomic and transcriptomic data indicated that the MLK4 gene (*MAP3K21/KIAA1804*) is frequently mutated and overexpressed in different types of human cancer [15]. Nonetheless, the distinct functions of MLK4 in cancer cell biology and tumor progression remain poorly understood. Intriguingly, the previous studies described both prooncogenic and tumor-suppressive functions of MLK4 [16]. It was demonstrated that MLK4 negatively regulates MAPK signaling pathways and impairs the invasive potential of ovarian cancer cells [17, 18]. Contrary to these findings, we and others showed that MLK4 directly phosphorylates MEK1 and MKK4/7 to activate ERK and JNK pathways in melanoma and colorectal cancer cells [19–21]. The study by Kim et al. indicated that MLK4 induces a mesenchymal phenotype and aggressive growth of glioblastoma cells in an NF-κB-dependent manner [22]. Similarly, we found that MLK4 activates the NF-κB pathway in TNBC, which leads to the high metastatic and invasive potential of breast cancer cells [23]. Here, we investigate the functional role of MLK4 in chemoresistance in TNBC. Our findings link MLK4 with DDR pathway activity upon treatment with DNA-damaging chemotherapy, and thus highlight MLK4 as an attractive target to combat chemoresistance.

## Materials and methods

### Cell lines and reagents

HCC1806, MDA-MB-436 cell lines were a kind gift from CRUK Manchester Institute, UK. SUM149PT cell line was purchased from BioIVT. HEK293T and MCF10A cell lines were purchased from ATCC. U2OS cell line was a kind gift from the National Cancer Institute, Frederick, MD, USA. HCC1806 cells were cultured in RPMI-1640 supplemented with 10% FCS, 1% penicillin/streptomycin, 2 mM L-glutamine and 1 mM sodium pyruvate. MDA-MB-436 cells were cultured in RPMI-1640 with 25 mM HEPES supplemented with 10% FCS, 1% penicillin/streptomycin, 2 mM L-glutamine, 1 mM sodium pyruvate and 10 µg/ml insulin. SUM149PT cells were cultured in Ham’s F12 medium with 25 mM HEPES supplemented with 10% FCS, 1% penicillin/streptomycin, 1 µg/ml hydrocortisone and 10 µg/ml insulin. HEK293T and U2OS cells were cultured in DMEM supplemented with 10% FCS, 1% penicillin/streptomycin, 2 mM L-glutamine and 1 mM sodium pyruvate. MCF10A cells were grown in DMEM/F12 supplemented with 5% horse serum, 20 ng/ml EGF, 0.5 mg/ml hydrocortisone, 100 ng/ml cholera toxin, 10 µg/ml insulin, 1% penicillin/streptomycin. Cell lines were authenticated by short tandem repeat profiling by ATCC Service at the beginning of the research and in December 2020. Cell lines were screened for mycoplasma regularly. Doxorubicin hydrochloride, etoposide, neocarzinostatin and KU-60019 were purchased from Sigma Aldrich. Recombinant human IL-6 was purchased from Peprotech. CEP-5214 was a kind gift from Dr. John Brognard, NCI, Frederick, MD, USA.

### siRNA and plasmid transfection

JetPRIME (Polyplus Transfections) was used for plasmids and siRNA transfections according to the manufacturer’s instructions. MLK4 silencing siRNA smart-pool (siRNA 1–4) and control siRNA (siGENOME Non-Targeting siRNA #5) were purchased from Dharmacon (Supplementary Table S1). MLK4-WT vector was obtained from GeneCopoeia, cloned and mutations (KA = kinase active and KD = kinase dead) were introduced as previously described [20]. pDRGFP and CBASceI vectors were gifts from Maria Jasin (Addgene plasmid # 26475 and # 26477) [24]. pimEJ5GFP was a gift from Jeremy Stark (Addgene plasmid # 44026) [25].

### AnnexinV apoptosis assay

Cells were transfected with MLK4-targeting siRNA or control siRNA and after 24 h doxorubicin and etoposide were added at indicated concentrations. After 48 h of treatment, cells were harvested and stained using the AnnexinV-FITC apoptosis detection kit (R&D Biosystems and Invitrogen), according to the manufacturer’s instructions. Flow cytometry analysis was performed with LSR II Fortessa (BD Biosciences).

### Cell viability assays

HCC1806 and SUM149PT doxycycline-inducible cell lines were seeded into six-well plates and incubated with 1 µg/ml doxycycline to induce MLK4 knock-down. MCF10A cells were transfected with either MLK4-targeting or control siRNA. Subsequently, cells were incubated with doxorubicin and etoposide at indicated concentrations. After 48 h of treatment, cells were fixed with 4% PFA and stained with 0.5% crystal violet solution prepared in 25% methanol. Wells were thoroughly washed and air-dried. For quantification, 2 ml of 10% acetic acid was added to each well, plates were incubated for 20 min with shaking and absorbance values were read at 590 nm using the microplate reader Synergy II (BioTek).

### Comet assay

Comet assays were performed using CometAssay Kit (Trevigen) following the manufacturer’s instructions. Pictures were taken with a Nikon Eclipse Fluorescent microscope. OPENCOMET plugin for Fiji was used for image analysis.

### Analysis of DSB repair by reporter assays

The efficacy of DNA double-strand break repair was measured using GFP-based reporter assays as described previously [24, 25]. Briefly, cells were transfected with either pDR-GFP or pimEJ5-GFP vector. The transfected cells were selected for puromycin resistance for at least two weeks. Selected cell lines were transfected with MLK4-targeting siRNA or control siRNA. The next day, cells were transfected with the SceI endonuclease expression vector (pCBA-SceI) to induce DSBs. 48 h after the induction of DSBs, cells were analyzed by flow cytometry (LSR II Fortessa (BD Biosciences). The percentage of GFP-positive cells was used as an indication of HR and NHEJ efficiency. pGFPMax vector (Lonza) was used as a positive control to monitor the transfection efficiency.

### Mouse xenografts and in vivo studies

All procedures were approved by the Local Ethics Committee at the University of Warsaw (1035/2020) and carried out in accordance with the requirements of EU (Directive 2010/63/EU) and Polish (Dz. U. poz. 266/15.01.2015) legislation. 8 to 14-week-old RAG2^-/-^ female mice were injected into mammary fat pads with 3 × 10^6^ HCC1806_sh6 cells with Matrigel in proportion 1:1. Mice were allocated randomly into cages and doxycycline was administered in drinking water one day after injections. Doxorubicin or equal volumes of 0.9% NaCl were administered intraperitoneally when the average tumor size reached 100 mm^3^. The next doses were administered at 5-day intervals. The tumor growth was monitored two times/week. Tumor volume was calculated based on caliper measurements, using the formula: tumor volume = (*D* × *d*^2^ × *π*)/6, where *D* is the bigger measurement, and *d* is the smaller measurement. Mice were culled after 4 weeks post injection. Tumors were resected, weighed, and processed for further analyses.

### Library preparation and next-generation sequencing

HCC1806 cells were transfected in triplicates with non-targeting control siRNA or MLK4-targeting siRNA. 24 h after transfection, cells were either left untreated or treated with 1 μM doxorubicin for additional 24 h. Next, RNA was isolated using the RNAeasy Mini Kit (Qiagen), according to the manufacturer’s instructions. Libraries were prepared using NEBNext Poly(A) mRNA Magnetic Isolation Module (New England Biolabs) and KAPA RNA HyperPrep Kit (Kapa Biosciences). The quality of the obtained libraries was tested using Bioanalyzer-2100 and High Sensitivity DNA kit (Agilent). Nucleic acid quantity in the libraries was measured by qPCR using the Kapa Library Quantification kit (Kapa Biosciences). Pair-end sequencing was performed with the NovaSeq 6000 S1 Reagent Kit (200 cycles, Illumina) using NovaSeq 6000 instrument (Illumina). mRNA-seq data processing is described in the Supplementary Information file. All mRNA-seq data have been deposited at GEO DataSets (GSE174692).

### Statistical analysis

All experiments were performed at least three times, unless otherwise indicated. For flow cytometry data analysis Flow Jo v10.6.1 software (TreeStar) or BD FACSDiva software (BD Biosciences) were used. Statistical analyses were carried out with GraphPad Prism 7 software. Data are expressed as the mean ± the standard error of the mean (SEM). P-value <0.05 was considered significant: *p < 0.05, **p < 0.01, ***p < 0.001, ****p < 0.0001. For the experiments where the comparison was performed between more than two groups, the statistical significance was assessed by one-way ANOVA followed by Tukey post-hoc tests or two-way ANOVA. For the experiments where two groups were compared, the statistical significance was determined by unpaired Student’s *t*-test. Analysis of the drugs’ interaction was performed using Combenefit software, as described previously [26].

The lists of antibodies and primers used in this study are included in Supplementary Table S2 and Supplementary Table S3, respectively. For additional methods, please see the Supplementary Information File.

## Results

### MLK4 loss or inhibition sensitizes TNBC cells to chemotherapy in vitro

**Fig. 1:**
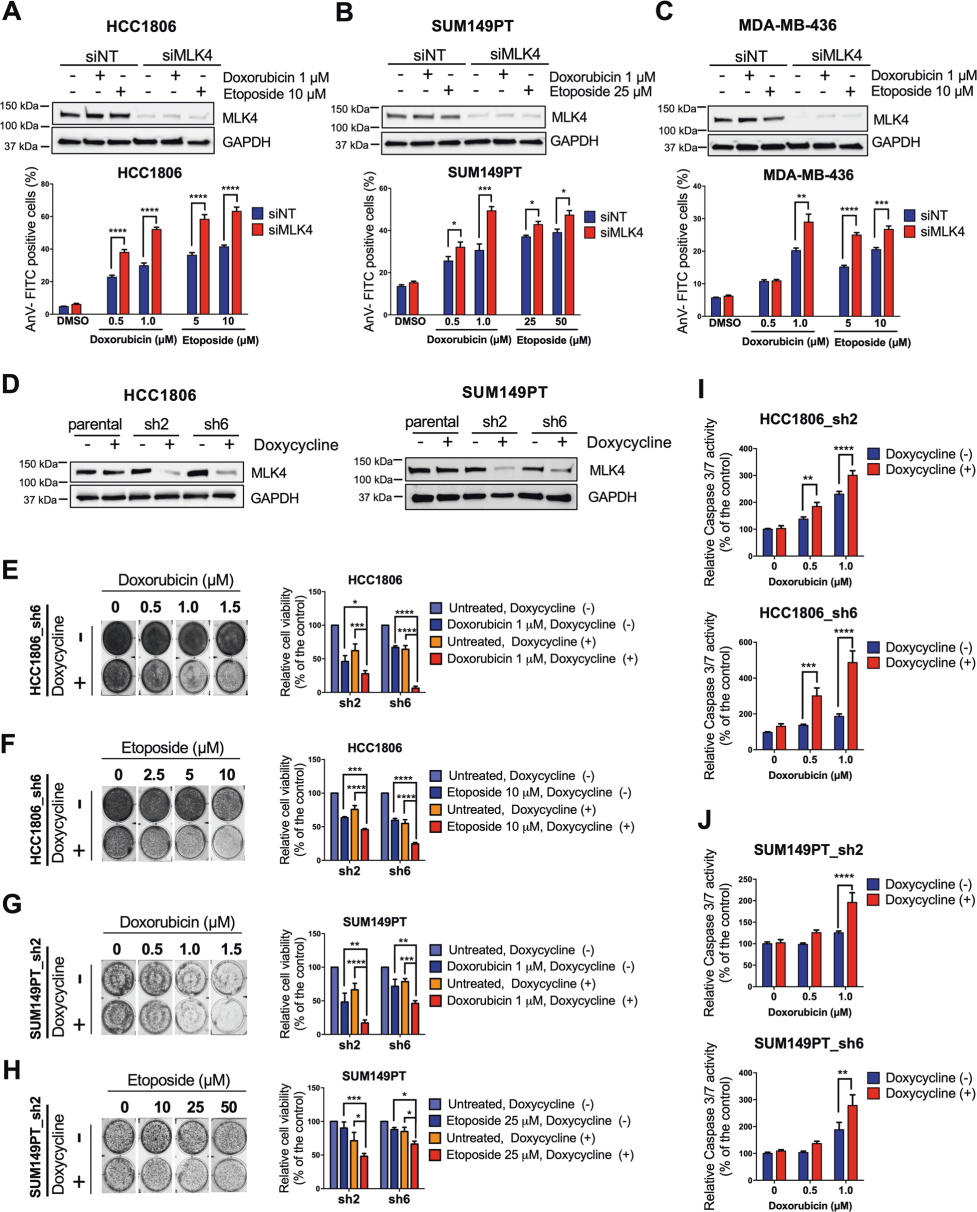
MLK4 knock-down increases sensitivity of triple-negative breast cancer cells to doxorubicin and etoposide. **A**–**C** HCC1806, SUM149PT and MDA-MB-436 cells were transfected using siRNA against MLK4 (siMLK4) or non-targeting siRNA control (siNT). After 24 h, cells were treated with doxorubicin and etoposide at indicated concentrations for an additional 48 h. Next, cells were stained with AnnexinV-FITC, and analyzed by flow cytometry. Error bars indicate ±SEM from two or three independent experiments, performed in triplicates. Significance was calculated using an unpaired two-tailed *t*-test, *p < 0.05, **p < 0.01, ***p < 0.001, ****p < 0.0001. **D** Cell lines with doxycycline-inducible MLK4 knock-down were generated from HCC1806 and SUM149PT parental cells using lentiviral vectors. Silencing of MLK4 was confirmed by immunoblotting. **E**–**H** HCC1806_sh2, HCC1806_sh6, SUM149PT_sh2 and SUM149PT_sh6 cell lines were incubated with doxycycline to induce MLK4 knock-down and subsequently cells were treated with doxorubicin and etoposide at indicated concentrations for 48 h. Following treatment, cells viability was assessed by crystal violet staining and quantified by absorbance measurements. Representative plates after crystal violet staining are shown on the left. Error bars indicate ±SEM from three independent experiments (*n* = 3). Significance was calculated using one-way ANOVA followed by Tukey multiple comparisons test, *p < 0.05, **p < 0.01, ***p < 0.001, ****p < 0.0001. **I**–**J** MLK4-silenced and control cells were treated with doxorubicin at increasing concentrations and the activity of caspases 3/7 was measured using bioluminescence assay. Error bars indicate ±SEM from three independent experiments, performed in triplicates (*n* = 9). Significance was calculated using an unpaired two-tailed *t*-test, **p < 0.01, ***p < 0.001, ****p < 0.0001.

To study the role of MLK4 in response to chemotherapeutic drugs treatment, we used TNBC cell lines with high endogenous expression of this kinase––HCC1806, SUM149PT, and MDA-MB-436 [23]. Cells were transfected with MLK4-targeting smart-pool or control siRNA and either left untreated or treated with increasing concentrations of topoisomerase II poisoning chemotherapeutics––doxorubicin and etoposide. Downregulation of MLK4 markedly increased apoptosis of TNBC cells upon treatment with both drugs, as revealed by the measurements of AnnexinV-positive cells using flow cytometry (Fig. 1A–C, Supplementary Fig. S1A–C). MLK4 knock-down did not cause apoptosis in untreated cells, which agrees with our previous observations, confirming that MLK4 loss alone is not sufficient to induce TNBC cells death [23]. To exclude the possible off-target effects of MLK4-targeting siRNA smart-pool, we validated MLK4 knock-down using each of the four individual siRNAs, which all decreased the viability of HCC1806 cells in the presence of doxorubicin (Supplementary Fig. S2A, B). To further study the involvement of MLK4 in TNBC chemoresistance, we generated HCC1806 and SUM149PT cell lines with doxycycline-inducible MLK4 knock-down system, using two different lentiviral shRNA vectors––sh2 and sh6 (Fig. 1D). Following MLK4 downregulation, both cell lines exhibited decreased viability in the presence of doxorubicin and etoposide (Fig. 1E–H). The reduction in cell viability resulted from chemotherapeutics-induced apoptosis, as indicated by the higher activity of caspases 3 and 7 in MLK4-depleted cells upon doxorubicin treatment (Fig. 1I, J). To exclude the possibility that the observed effects are related to the use of doxycycline itself, we treated parental cell lines, HCC1806 and SUM149PT, with doxycycline and chemotherapeutics. Incubation with doxycycline did not decrease the viability of HCC1806 and SUM149PT parental cell lines upon treatment with doxorubicin or etoposide (Supplementary Fig. S3A-D) and did not increase apoptosis induction in response to chemotherapy (Supplementary Fig. S3E, F), suggesting that the observed effects were due to MLK4 silencing. Conversely to MLK4 knock-down, the induced overexpression of this kinase led to an increased viability of HCC1806 cells following doxorubicin treatment (Supplementary Fig. S4A, B), indicating that high MLK4 expression protects TNBC cells from chemotherapy-induced cell death.

**Fig. 2:**
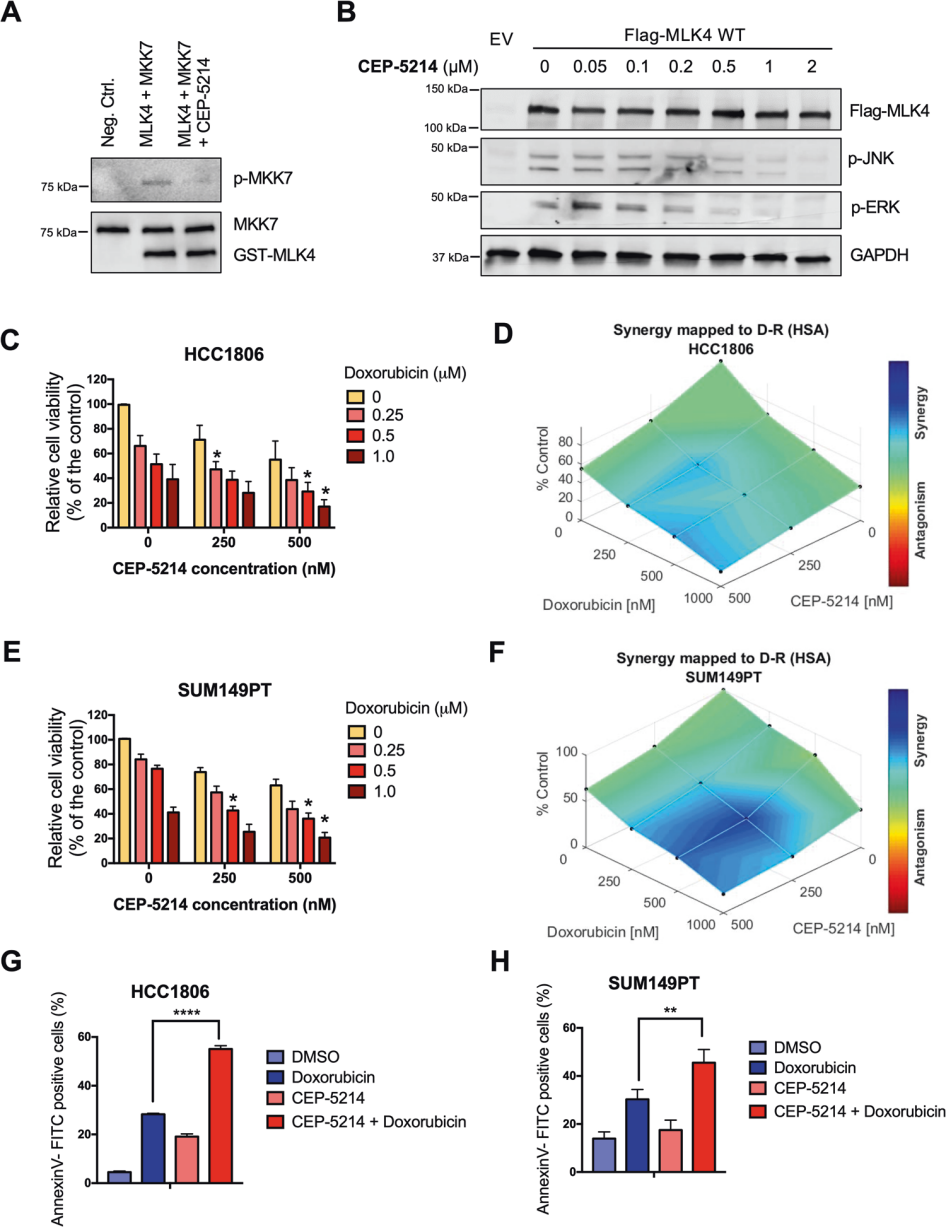
MLK4 inhibition sensitizes TNBC cells to chemotherapy. **A** Kinase-inactive MKK7 and purified GST-MLK4 kinase domain (isolated from baculovirus-infected insect cells) were subjected to in vitro kinase assay in the presence or absence of CEP-5214 inhibitor. **B** HEK293T cells were transiently transfected with MLK4-WT vector or with the empty vector and incubated with increasing concentrations of CEP-5214 for 1 h. Next, whole cell lysates were collected and analyzed by immunoblotting. **C**–**F** HCC1806 (C-D) and SUM149PT (E-F) cells were incubated with CEP-5214 or DMSO for 72 h and doxorubicin for 48 h. After treatment, cells viability was assessed by crystal violet staining and quantified by absorbance measurements. Error bars indicate ±SEM from three independent experiments. Analysis of combination efficacy and synergy was performed using HSA model with Combenefit software, *p < 0.05. **G**–**H** HCC1806 and SUM149PT cells were incubated with CEP-5214 at concentrations 500 nM and 250 nM, respectively or DMSO for 72 h and doxorubicin for 48 h. Next, cells were stained with AnnexinV-FITC, and measured by flow cytometry. Error bars indicate ±SEM from two independent experiments, performed in triplicates. Significance was calculated using one-way ANOVA followed by Tukey multiple comparisons, **p < 0.01, ****p < 0.0001.

Following our observation that knock-down of MLK4 increased sensitivity of TNBC cell lines to chemotherapy, we aimed to investigate if pharmacological inhibition of this kinase could also improve the efficacy of doxorubicin against TNBC cells. Since no small molecule inhibitors specifically inhibiting MLK4 kinase have been described so far, we studied other commercially available compounds that could be used to target MLK4-mediated signaling. CEP-5214, a pan-VEGF-R and MLK1-3 inhibitor [27], showed potent inhibitory activity against MLK4, as demonstrated by an in vitro kinase assay and further confirmed by the inhibition of ERK and JNK phosphorylation in HEK293T cells transiently transfected with MLK4 (Fig. 2A, B). Notably, treatment with CEP-5214 potentiated doxorubicin cytotoxicity against HCC1806 and SUM149PT cell lines, and this effect was synergistic (Fig. 2C–F). The combination treatment significantly enhanced apoptosis induction in both cell lines tested, as compared to the treatment with doxorubicin and CEP-5214 alone (Fig. 2G, H). These data confirm that MLK4 is a druggable kinase and provide evidence that depletion or inhibition of MLK4 sensitizes TNBC cells to chemotherapy. We next tested whether the combination of MLK4 silencing and chemotherapy affects non-malignant cells. Knock-down of MLK4 in breast epithelial cell line MCF10A, which is characterized by relatively low endogenous expression of this kinase, did not result in a decreased cell viability in the presence of doxorubicin and etoposide (Supplementary Fig. S5A, B). Furthermore, MLK4 silencing did not increase apoptosis induction and caspases 3/7 activation in MCF10A cells upon treatment with chemotherapy (Supplementary Fig. S5C, D). Finally, treatment with CEP-5214 did not enhance doxorubicin cytotoxicity against MCF10A cells (Supplementary Fig. S5E, F). These results indicated that MLK4 loss or inhibition did not potentiate the toxic effects of chemotherapy against normal cells.

### MLK4 knock-down increases chemosensitivity of TNBC cells in 3D cell culture and in vivo models

**Fig. 3:**
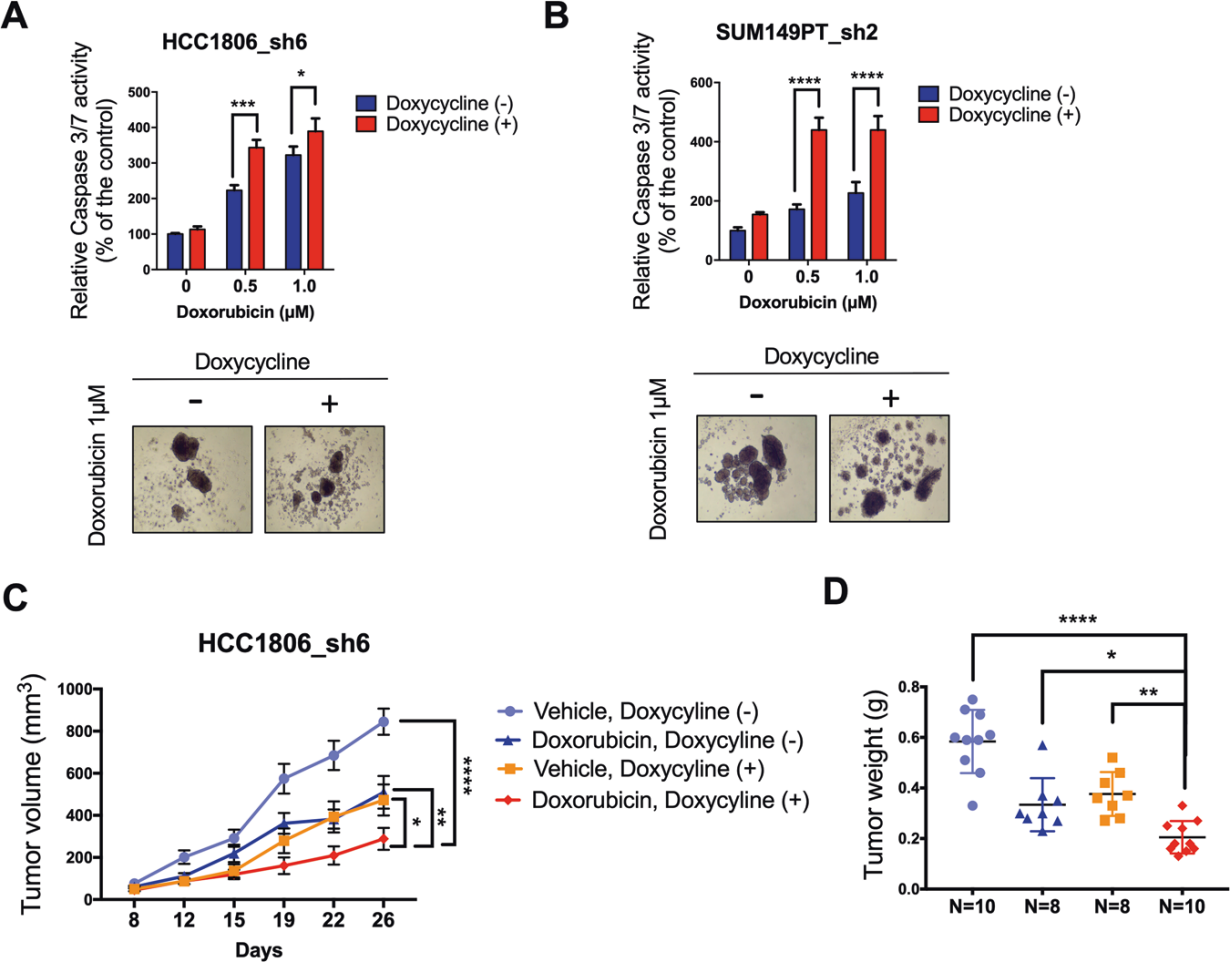
MLK4 promotes chemoresistance in 3D cell culture and in vivo models. **A**–**B** MLK4 knock-down was induced by doxycycline in HCC1806_sh6 and SUM149PT_sh2 cells grown in non-adherent conditions and subsequently cells were treated with doxorubicin at indicated concentrations for 48 h. The activity of caspases 3/7 was measured using bioluminescence assay. Representative pictures of mammospheres upon doxorubicin treatment are shown. Error bars indicate ±SEM from three independent experiments, performed in triplicates (*n* = 9). Significance was calculated using an unpaired two-tailed *t*-test, *p < 0.05, **p < 0.01, ****p < 0.0001. **C** HCC1806_sh6 cells were injected into mammary fat pads of RAG2^−/−^ mice. Doxycycline was administered one day after the injection to induce MLK4 knock-down. Mice were treated with doxorubicin (4 mg/kg, i.p.) or saline at 5-day intervals, starting from day 7 of the experiment. Tumors were measured twice a week. Error bars indicate ±SEM (*n* = 10 for control group, *n* = 8 for doxorubicin-treated group, *n* = 8 for doxycycline-treated group, *n* = 10 for combination treatment group). Statistical comparison was performed using two-way ANOVA, *p < 0.05, **p < 0.01, ****p < 0.0001. **D** Weight of tumors resected at the end of the study. Significance was calculated using one-way ANOVA followed by Tukey multiple comparisons test, *p < 0.05, **p < 0.01, ****p < 0.0001.

3D-cultured breast cancer cells forming mammospheres reflect tumor growth in vivo more closely than traditional two-dimensional culture, and therefore, may provide more accurate models to study cancer biology and therapy [28]. Thus, we tested if MLK4 loss could sensitize TNBC cells grown as mammospheres to doxorubicin treatment. We observed increased activation of caspases 3 and 7 in mammospheres formed by MLK4-depleted HCC1806_sh6 and SUM149PT_sh2 cells treated with doxorubicin (Fig. 3A, B). Next, we used a xenograft-based approach to test the effects of MLK4 loss on doxorubicin sensitivity of TNBC tumors in vivo. Consistent with our previous results, the growth of HCC1806_sh6 xenograft tumors was abrogated by MLK4 silencing [23]. The combination of MLK4 knock-down with doxorubicin treatment showed a further significant reduction in tumor growth (Fig. 3C, D). Taken together, our data suggest that MLK4 loss introduces a therapeutic vulnerability by sensitizing tumors towards cytotoxic chemotherapy in vivo.

### MLK4 depletion leads to an increased accumulation of doxorubicin-induced DNA damage in TNBC cells

**Fig. 4:**
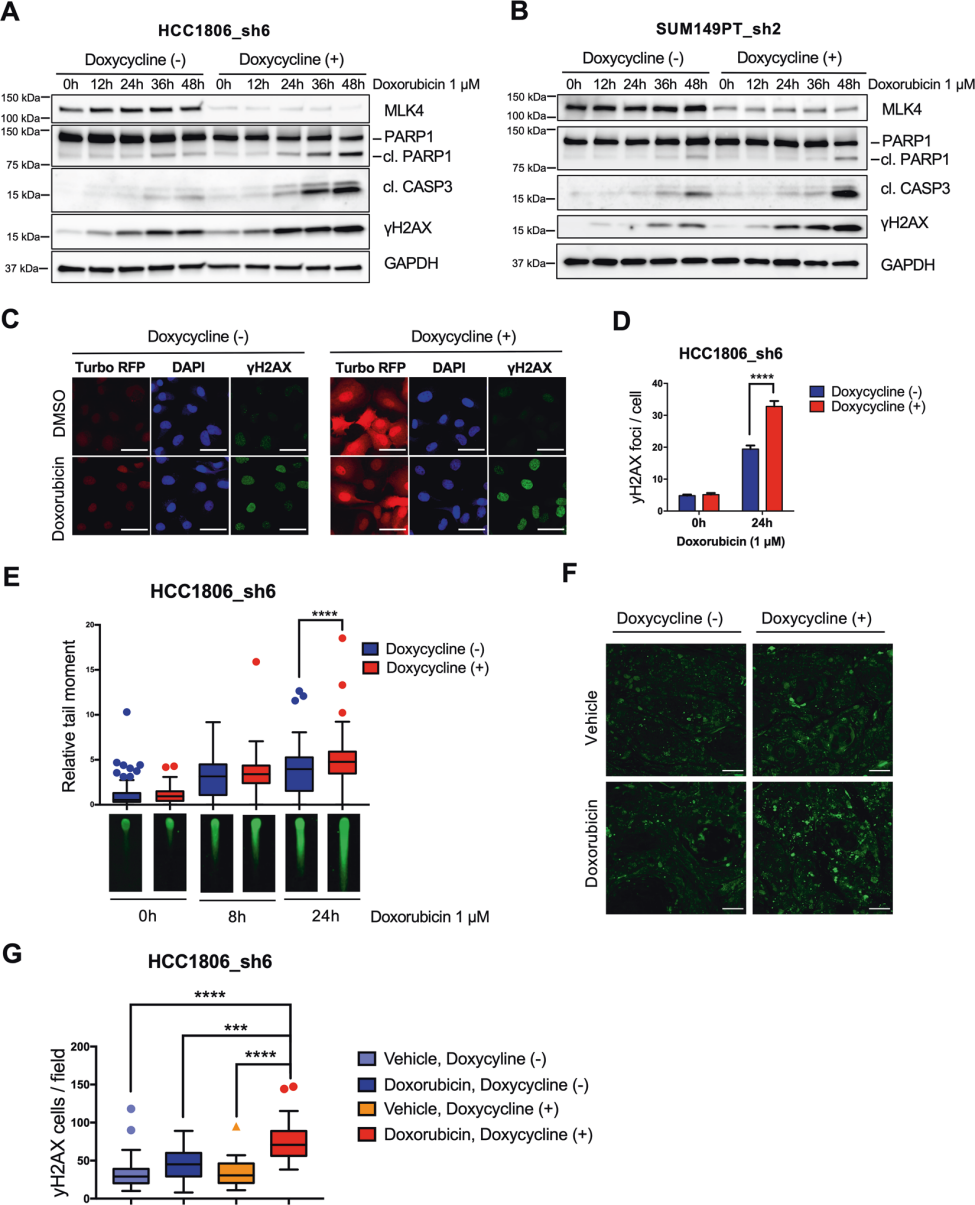
MLK4 loss results in an accumulation of doxorubicin- induced DNA damage in TNBC cells. **A**–**B** MLK4 knock-down was induced by doxycycline in HCC1806_sh6 and SUM149PT_sh2 and subsequently cells were treated with doxorubicin for the indicated time. Following the treatment, whole cell lysates were collected and analyzed by immunoblotting. The persistent phosphorylation of γH2AX in MLK4-depleted cells was associated with apoptotic cell death, as suggested by caspase 3 activation and PARP1 cleavage. **C** HCC1806_sh6 cells were incubated with doxycycline to induce MLK4 knock-down, treated with doxorubicin for 24 h and stained for γH2AX [green] and DAPI [blue]. The expression of turbo-RFP [red] is induced along with the expression of MLK4-targeting shRNA. Scale bars 50 μm. **D** Nuclear foci of γH2AX were quantified in at least 50 cells in each group per experiment using FIJI. Error bars indicate ±SEM from three independent experiments. Significance was calculated using an unpaired two-tailed *t*-test, ****p < 0.0001. **E** HCC1806_sh6 cells were incubated with doxycycline to induce MLK4 knock-down and treated with doxorubicin for 8 h or 24 h. After the treatment, the extent of DNA damage was analyzed by neutral comet assay. Relative comet tail moment was quantified in at least 50 cells in each group per experiment using ImageJ Open Comet plugin. Error bars indicate ±SEM from three independent experiments. Significance was calculated using an unpaired two-tailed *t*-test, ****p < 0.0001. **F** HCC1806_sh6 xenograft tumors were harvested and stained for γH2AX [green]. Scale bars 50 μm. **G** γH2AX-positive cells in tumor sections were quantified using FIJI. Significance was calculated using one-way ANOVA followed by Tukey multiple comparisons test, ***p < 0.001, ****p < 0.0001.

The major mechanism of action of topoisomerase II poisons involves the induction of DNA double-strand breaks (DSB) that, if not repaired, result in cell death [29]. Formation of the DNA DSBs induces phosphorylation of H2AX at Ser139 (γH2AX), which is a sensitive biomarker for DNA damage [30]. We found that MLK4-deficient HCC1806_sh6 and SUM149PT_sh2 cell lines showed increased H2AX phosphorylation after 24–48 h of treatment with doxorubicin (Fig. 4A, B), as well as enhanced formation of doxorubicin-induced nuclear γH2AX foci (Fig. 4C, D). MLK4 knock-down also resulted in significantly higher levels of doxorubicin-induced DNA DSBs as determined by comet assay (Fig. 4E). Finally, the analysis of HCC1806_sh6 xenograft tumors by immunofluorescent staining revealed that MLK4 knock-down was associated with persistent H2AX phosphorylation in tumor tissues after the treatment with doxorubicin in vivo (Fig. 4F, G). Collectively, these results indicate that MLK4 loss leads to excessive accumulation of DNA damage induced by chemotherapeutics and contributes to cancer cells death and chemosensitivity.

### MLK4 regulates ATM activation and downstream DNA damage response signaling in TNBC cells

**Fig. 5:**
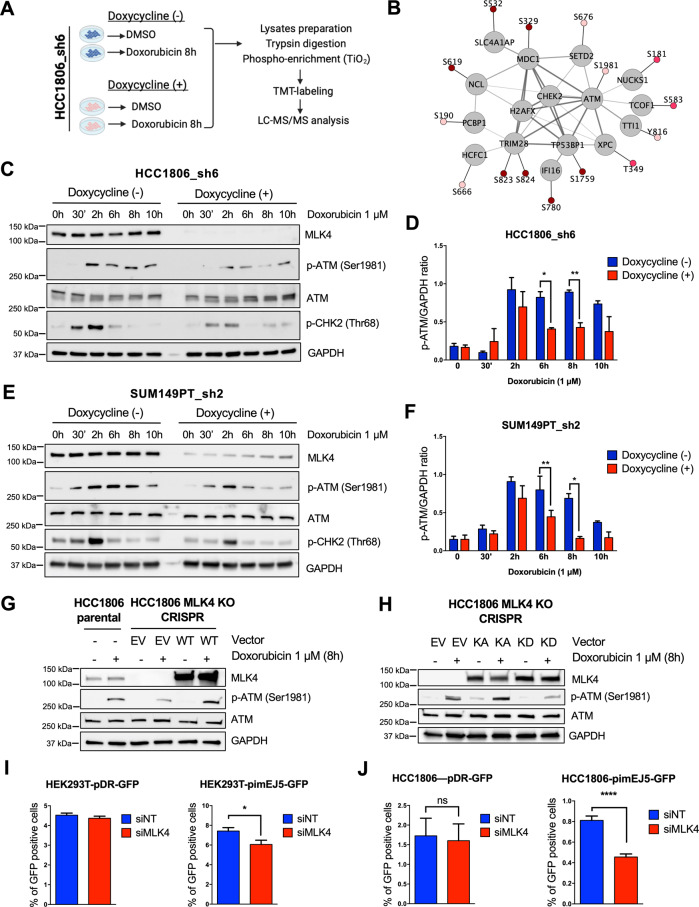
MLK4 knock-down impairs ATM activation and DNA damage repair in TNBC cells. **A** Workflow summary for multiplexed, quantitative phosphoproteomic analysis of HCC1806_sh6 cells. MLK4 knock-down was induced by the addition of doxycycline to the cell culture medium. Next, cells were treated with doxorubicin or DMSO for 8 h. Following the treatment, whole cell lysates were collected, samples were digested and alkylated prior to phosphopeptide enrichment on TiO2 beads, addition of 10-plex tandem mass tag (TMT) labels and analysis of phosphopeptides using LC-MS/MS. **B** Network of selected proteins related to DNA damage with phosphosites increasing upon doxorubicin in control (*q* < 0.15, positive log2 [fold-change]) but not in MLK4-depleted HCC1806_sh6 cells. Color fills represent the significance of the change in control cells upon doxorubicin treatment (dark red: *q* < 0.05, red: *q* = 0.05–0.1, light red: *q* = 0.1–0.15). **C**–**F** MLK4 knock-down was induced by doxycycline in HCC1806_sh6 and SUM149PT_sh2 cells, which were next treated with doxorubicin for the indicated time. Following the treatment, whole cell lysates were collected and analyzed by immunoblotting. Densitometry and quantitative analysis of immunoblotting results was performed from at least 3 independent experiments. GAPDH was used to normalize the band’s intensity. Error bars indicate ±SEM. Significance was calculated using an unpaired two-tailed *t*-test, *p < 0.05, **p < 0.01. **G** HCC1806 CRISPR-MLK4 knock-out cells were transfected with MLK4-WT vector to rescue MLK4 expression or with the empty vector. Following the transfection, HCC1806 CRISPR-MLK4 knock-out cells and HCC1806 parental cells were treated with doxorubicin for 8 h. After the treatment, whole cell lysates were collected and analyzed by immunoblotting. **H** HCC1806 CRISPR-MLK4 knock-out cells were transfected with MLK4 KA (kinase active), MLK4 KD (kinase dead) and control empty vector (EV). Following the transfection, cells were treated with doxorubicin for 8 h. Next, whole cell lysates were collected and analyzed by immunoblotting. **I–J** HEK293T-pDR-GFP, HEK293T-piMEJ5-GFP, HCC1806-pDR-GFP and HCC1806-pimEJ5-GFP cell lines were generated from parental cells lines by transfection of the appropriate vectors and subsequent selection with puromycin for over 14 days. The cells stably expressing reporter vectors were transfected with MLK4-targeting siRNA (siMLK4) or control non-targeting siRNA (siNT) along with pSCE-CbaI endonuclease expressing vector. After 72 h, the activity of homologous recombination (pDR-GFP vector expressing cells) and non-homologous end joining (pimEJ5-GFP vector expressing cells) DNA repair pathways was assessed by measuring the percentage of GFP-positive cells using flow cytometry. Error bars indicate ±SEM from three independent experiments performed in triplicates (*n* = 9). Significance was calculated using an unpaired two-tailed *t*-test, *p < 0.05, ****p < 0.0001.

Next, to determine cellular mechanisms responsible for the identified MLK4-dependent chemoresistance in TNBC, we performed a quantitative phosphoproteomic analysis of control and MLK4-depleted cells, either untreated or treated with doxorubicin (Fig. 5A). We found that the doxorubicin-induced phosphorylation of several core DNA damage response components, including ATM, TRIM28 (KAP-1), MDC1 and TP53BP1, was impaired in cells lacking MLK4 (Fig. 5B, Supplementary Table S4). Western blot analysis of HCC1806_sh6 and SUM149PT_sh2 cells exposed to doxorubicin at different time points confirmed that ATM phosphorylation at Ser1981 was diminished upon MLK4 knock-down, particularly after 6–8 h of treatment with doxorubicin (Fig. 5C–F). Additionally, we observed a decreased phosphorylation of CHK2, an ATM downstream effector kinase, suggesting that MLK4 knock-down compromised ATM signaling in TNBC cells (Fig. 5C, E). These observations were validated by the analysis of protein lysates from HCC1806_sh6 and SUM149PT_sh2 cells treated with DNA DSBs-inducing radiomimetic agent––neocarzinostatin (NCS), which also revealed a decreased phosphorylation of ATM after 6 h of treatment in MLK4-deficient cells (Supplementary Fig. S6A, B). To confirm the involvement of MLK4 in ATM activation, we generated HCC1806 cells with a permanent CRISPR/Cas9-mediated deletion of the MLK4 gene. Consistent with shRNA-mediated knock-down, HCC1806 cells with MLK4 knock-out showed decreased ATM activation after the treatment with doxorubicin (Fig. 5G). At the same time, ATM phosphorylation at Ser1981 was completely rescued by the overexpression of MLK4 wild-type protein in these cells (Fig. 5G). Furthermore, the observed effect is likely to be kinase-dependent, as overexpression of MLK4 kinase-active mutant, but not MLK4 kinase-dead mutant, increased phosphorylation of ATM in MLK4 knock-out cells (Fig. 5H).

DNA DSBs are repaired by two major pathways, namely by non-homologous end joining (NHEJ) and homologous recombination (HR). ATM signaling has been shown to coordinate these DNA repair pathways and modulate their activity at multiple stages in a context-dependent manner [31, 32]. Thus, we sought to determine whether MLK4 knock-down impairs the repair of DSBs by one or both DNA repair pathways. We generated HEK293T and HCC1806 cell lines harboring plasmid-based GFP reporter specific for HR- or NHEJ-mediated DSBs repair (pDR-GFP and pimEJ5-GFP, respectively) [24, 25]. Using these cell lines, we were able to evaluate the efficiency of HR and NHEJ based on the number of GFP positive cells measured by flow cytometry. We found that MLK4 knock-down decreased the efficiency of NHEJ-mediated DSBs repair but only slightly reduced HR-mediated DNA repair efficiency in both cell lines (Fig. 5I, J). Although HCC1806 cells do not harbor deleterious BRCA1/2 mutations and our reporter assays suggested that they are proficient in performing HR-mediated DSBs repair, previous studies reported their sensitivity to some PARP inhibitors [33, 34]. This raises the possibility that HR might be at least partially impaired in this TNBC cell line due to defects other than BRCA1/2 mutations. Therefore, we validated our reporter assays results using additional cancer cell line model – U2OS, that does not have deleterious mutations in p53 and BRCA1/2 genes and is characterized by the largely intact function of the two major DNA DSBs repair pathways [35]. Using U2OS-pDR and U2OS-pimEJ5 cells, we further showed that MLK4 knock-down led to a decreased NHEJ-mediated DSBs repair without significantly affecting HR repair efficiency (Supplementary Fig. S7A, B). Taken together, our findings indicate that MLK4 is required for ATM activation and efficient DSBs repair, primarily via NHEJ pathway.

### MLK4 is required for DNA damage-induced transcriptional activation of NF-кB target genes

To gain additional insights into the molecular mechanisms involved in MLK4-mediated chemoresistance, we performed gene expression profiling by mRNA-seq in HCC1806 cells transfected with control or MLK4-targeting siRNAs and treated with either doxorubicin for 24 h or vehicle only. Principal component analysis (PCA) confirmed a high degree of reproducibility of our mRNA-seq data and showed that both MLK4 knock-down and doxorubicin treatment led to significant transcriptome-wide alterations in HCC1806 cells (Supplementary Fig. S8A). Differential gene expression analysis revealed that approximately 25% of transcripts regulated by doxorubicin were unique to either MLK4-depleted or control cells, which suggested that MLK4 knock-down affects global transcriptomic changes in response to doxorubicin treatment (Supplementary Fig. S8B).

**Fig. 6:**
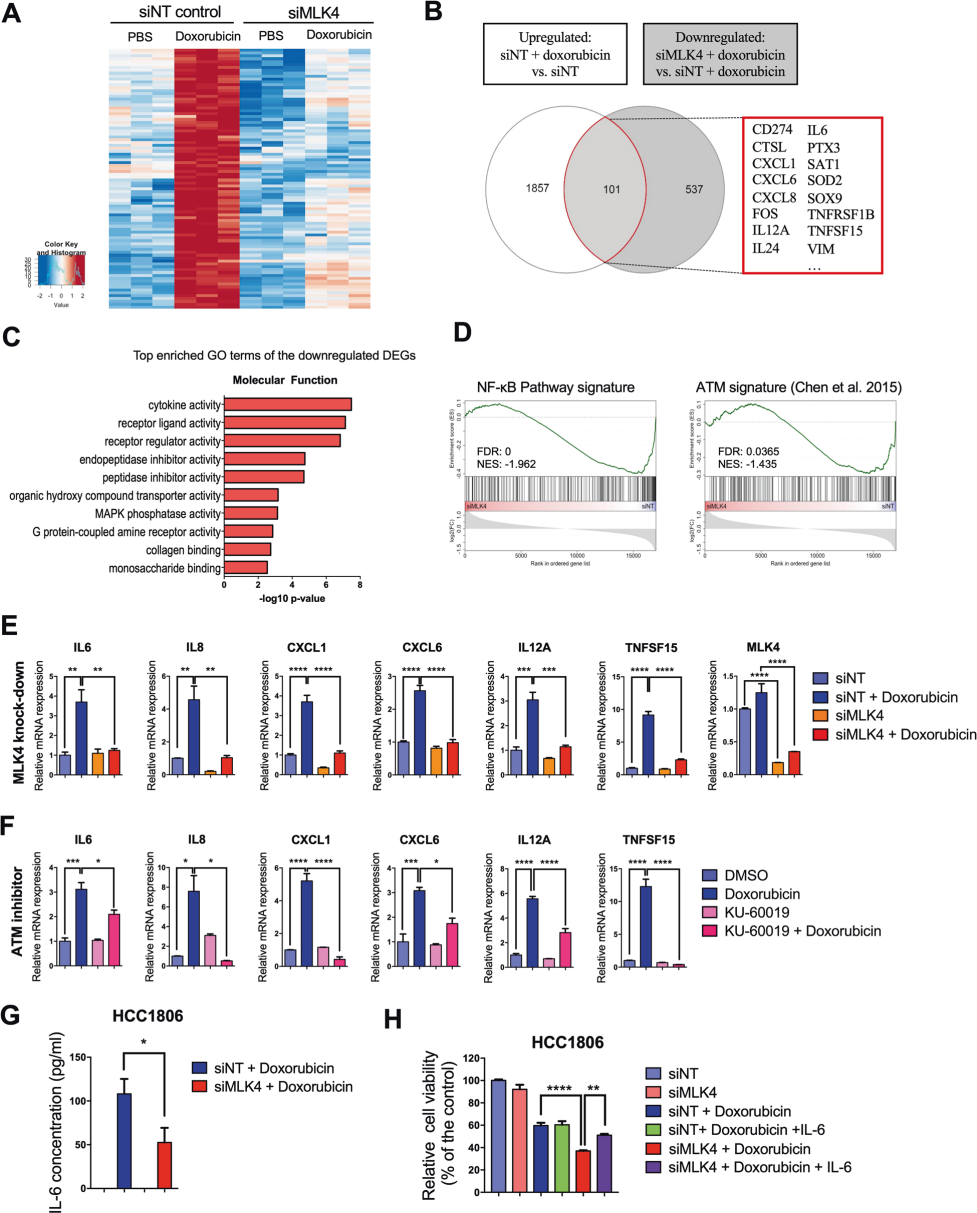
MLK4 loss interferes with the induction of NF-κB target genes and compromises adaptive transcriptional response of TNBC cells to chemotherapy. **A** Heatmap depicting the transcriptome-wide effects of MLK4 depletion and doxorubicin treatment. Shown are significantly [pvalue adj.<0.01] upregulated [log2 FC > 0.75, red] or downregulated [log2 FC < 0.75, blue] genes. **B** Venn diagram comparing the overlapping genes which are upregulated after doxorubicin treatment and significantly downregulated in MLK4-depleted cells compared to control cells after treatment with doxorubicin. The genes whose induction by doxorubicin is reduced by MLK4 loss are highlighted on the right. **C** GO enrichment analysis in MLK4-depleted vs. control cells treated with doxorubicin. **D** GSEA in MLK4-depleted vs. control cells treated with doxorubicin. The list of ATM-dependent genes was curated based on RNA-seq data from Chen et al. [37]. **E** HCC1806 cells were transfected with MLK4-targeting siRNA (siMLK4) or control non-targeting siRNA (siNT). Following the transfection, cells were treated with doxorubicin for 24 h. After the treatment, RNA was isolated and relative gene expression of several NF-κB target genes was analyzed by qRT-PCR. Error bars indicate ±SEM from three experiments (*n* = 3). Significance was calculated using one-way ANOVA followed by Tukey multiple comparisons test, **p < 0.01, ***p < 0.001, ****p < 0.0001. **F** HCC1806 cells were pre-treated with ATM inhibitor KU-60019 or DMSO for 1 h, and subsequently cells were treated with doxorubicin for 24 h. After the treatment, RNA was isolated and relative gene expression of indicated NF-κB target genes was analyzed by qRT-PCR. Error bars indicate ±SEM from three experiments. Significance was calculated using one-way ANOVA followed by Tukey multiple comparisons test, *p < 0.05, **p < 0.01, ***p < 0.001, ****p < 0.0001. **G** Following the transfection with either MLK4-targeting (siMLK4) or non-targeting control siRNA (siNT), cells were treated with doxorubicin for 24 h and the concentration of IL-6 in cell culture medium supernatants was measured using ELISA. Error bars indicate ±SEM from three experiments. Significance was calculated using an unpaired two-tailed *t*-test, *p < 0.05. **H** MLK4-depleted and control cells were incubated with doxorubicin alone (0.5 μM) or doxorubicin and IL-6 (10 ng/ml) for 48 h. Following the treatment, cells viability was assessed by crystal violet staining and quantified by absorbance measurements. Error bars indicate ±SEM from three experiments performed in triplicates (*n* = 9). Significance was calculated using one-way ANOVA followed by Tukey multiple comparisons test, **p < 0.01, ****p < 0.0001.

When comparing the overlap of transcripts that were upregulated by doxorubicin treatment in control cells (upregulated: siNT + doxorubicin versus siNT) with transcripts whose induction was reduced by MLK4 loss (downregulated: siMLK4 + doxorubicin versus siNT + doxorubicin), we identified a subset of 101 differentially regulated genes (Fig. 6A, B). Among these genes, we found numerous NF-κB targets, including several cytokines and paracrine factors. Functional enrichment analysis of genes downregulated following MLK4 depletion in doxorubicin-treated cells identified cytokine activity and receptor signaling as key downstream targets of MLK4 (Fig. 6C). Furthermore, Gene Set Enrichment Analysis (GSEA) showed that NF-κB signature was significantly downregulated in MLK4-depleted cells upon doxorubicin treatment (Fig. 6D). ATM activation and subsequent phosphorylation of its downstream substrates have been shown to play a pivotal role in adaptive transcriptional response following DNA damage [7, 36–38]. Thus, we curated a signature of ATM-dependent transcriptional alterations based on the previously published expression data from ATM-depleted TNBC cells [37]. Interestingly, we observed that genes previously found to be downregulated by ATM depletion were also negatively enriched in MLK4-deficient cells upon doxorubicin treatment in our experiments (Fig. 6D). To validate mRNA-seq results, we analyzed the expression of selected genes in MLK4-silenced and control HCC1806 cells by qRT-PCR. In agreement with our mRNA-seq data, the expression of several NF-κB-regulated cytokines (*IL-6, IL-8, CXCL1, CXCL6, IL-12A, TNFSF15*) was induced upon the treatment with doxorubicin in control cells, while the induction of these genes was compromised by MLK4 depletion (Fig. 6E). To establish whether ATM activation was necessary for the upregulation of the indicated NF-κB target genes by doxorubicin, we examined the expression of selected cytokines in HCC1806 cells treated with ATM inhibitor (KU-60019), doxorubicin or a combination of both. ATM inhibition led to a similar dampening of doxorubicin-induced transcriptional changes as MLK4 silencing (Fig. 6F), suggesting that the impaired induction of NF-κB target genes in MLK4-deficient cells is associated with a compromised ATM activation. Moreover, treatment with KU-60019 significantly enhanced doxorubicin cytotoxicity against HCC1806 and SUM149PT cells (Supplementary Fig. S9A, B).

ATM regulates NF-κB transcription in response to DNA damage through the phosphorylation of NEMO at Ser85, which aids in transmitting nuclear ATM signaling to activate NF-κB in the cytoplasm [39]. Upon activation, NF-κB-p65 translocates to the nucleus, where it regulates gene expression and promotes cell survival [40]. In agreement with our gene expression analyses, we observed reduced NEMO phosphorylation and decreased NF-κB-p65 nuclear translocation in MLK4-depleted cells upon treatment with doxorubicin (Supplementary Fig. S9C, D). Collectively, our results indicate that MLK4 is required for ATM activation that leads to downstream phosphorylation of NEMO and subsequent induction of NF-κB nuclear translocation in response to DNA damage.

### MLK4 loss impairs secretion of the pro-survival IL-6 in response to DNA-damaging chemotherapy

Our mRNA-seq data revealed that MLK4 knock-down diminished the doxorubicin-induced upregulation of several cytokines, which have been previously described to promote drug resistance in an autocrine/paracrine manner, including IL-6, IL-8 and CXCL1 [41–43]. Since IL-6-mediated autocrine signaling has been recently linked to multidrug resistance in TNBC, we verified if reduced IL-6 gene expression upon MLK4 knock-down contributes to increased chemosensitivity of HCC1806 cells. We confirmed a decreased secretion of IL-6 cytokine in MLK4-deficient cells upon treatment with doxorubicin using ELISA (Fig. 6G). Furthermore, we found that the addition of exogenous IL-6 to the cell culture medium increased viability of MLK4-depleted cells upon doxorubicin treatment, thus partially rescuing the effects of MLK4 knock-down (Fig. 6H). Together, these findings suggest that MLK4 function is required for the expression of NF-κB target genes to promote the survival of TNBC cells following chemotherapy.

### High expression of MLK4 is associated with poor prognosis in patients receiving anthracycline-based neoadjuvant chemotherapy

**Fig. 7:**
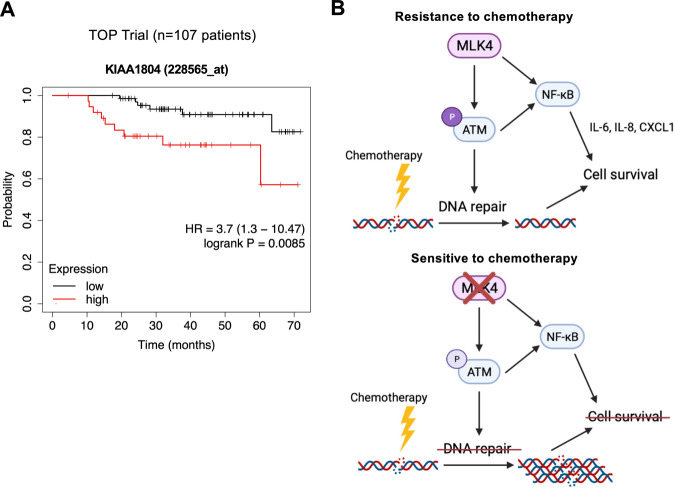
High MLK4 expression predicts poor prognosis in patients treated with anthracycline-based neoadjuvant chemotherapy. **A** Probability of overall survival in breast cancer patients from neoadjuvant TOP trial based on MLK4 mRNA expression in tumor tissue, evaluated using pre-treatment biopsies. Patients with estrogen receptor (ER)-negative tumors were treated with anthracycline (epirubicin) monotherapy. The OS was analyzed using the KMplotter tool, with auto-selected best cutoff. Graphic illustrations were obtained from Kmplot.com. **B** Schematic illustration of the proposed role of MLK4 in TNBC chemoresistance.

Finally, we aimed to explore the prognostic impact of high MLK4 gene expression in TNBC patients treated with neoadjuvant chemotherapy. We analyzed the gene expression and survival data from the TOP trial, in which patients with ER-negative tumors were treated with anthracycline monotherapy [44]. Our analysis indicated that high MLK4 mRNA expression in pre-treatment biopsies was associated with worse overall survival (Fig. 7A). These results suggest that high MLK4 expression in tumor tissue could be related to an unfavorable response to NAC and poor prognosis in TNBC patients.

## Discussion

The treatment of TNBC is limited by a lack of actionable targets and aggressive phenotype that is often refractory to cytotoxic chemotherapy. We recently found that MLK4 gene is amplified and highly expressed in the TNBC clinical samples and cell line models [23]. We also demonstrated that this kinase promotes proliferation, migration, and invasive potential of breast cancer cells [23]. However, the functional role of MLK4 in cancer chemoresistance has not been previously investigated. Here, we report our findings supporting the involvement of MLK4 in resistance of TNBC to clinically used chemotherapeutic agents. We demonstrated that MLK4 loss enhanced apoptosis induction and reduced viability of TNBC cell lines upon treatment with doxorubicin and etoposide in vitro, and these results were further recapitulated in breast cancer xenograft model. Notably, MLK4 knock-down alone did not cause cell death or DNA damage accumulation, which agrees with our previous data [23]. These observations provide a rationale for using MLK4 inhibitors in combination with DNA-damaging chemotherapies in the treatment of TNBC.

ATM is the primary kinase involved in the cellular response to DNA DSBs, and its loss or inhibition sensitizes cancer cells to chemotherapy [10, 14]. Recently, Colomer et al. described that ATM is directly activated by the nuclear form of IKKα kinase downstream of BRAF and TAK1, and inhibition of both IKKα and BRAF impaired DNA repair mechanisms, sensitizing metastatic tumors to DNA-damaging agents in vivo [11]. These results highlight the importance of upstream kinases in DDR regulation and point towards novel therapeutic strategies for overcoming cancer chemoresistance. We showed that MLK4 loss compromises ATM activation and inhibits phosphorylation of several DDR components, including: CHK2, KAP1, TP53BP1 and MDC1. Moreover, we demonstrated that MLK4 depletion attenuates downstream DNA repair via the NHEJ pathway, which seems to be the major pathway involved in repairing doxorubicin-induced DNA DSBs [45]. Thus, we propose that MLK4 plays a role in ATM activation to facilitate efficient DNA repair and contributes to TNBC survival and chemoresistance. There are several possible mechanisms through which MLK4 may impact ATM activation. MLK4 can directly interact with ATM and phosphorylate its residues, including Ser1981 or other phosphorylation sites essential for ATM activation [11, 46]. Alternatively, additional kinases downstream of MLK4 might be involved. For instance, IKKα/β, which are MLK4 substrates, were demonstrated to phosphorylate ATM in response to DNA damage and promote NHEJ-mediated DNA repair [11, 47]. Therefore, future studies are needed to elucidate the exact mechanisms by which MLK4 regulates ATM function and DDR signaling networks.

Currently, many drugs targeting various DDR components are in preclinical and clinical development. PARP inhibitors have been established as important new therapies for breast cancer patients with inherited BRCA1/2 mutations [48]. ATM inhibitors are now under investigation in phase I clinical trials in patients with glioblastoma and other advanced tumors in combination with radio- and chemotherapies [49]. However, the potential side-effects of prolonged treatment with ATM inhibitors should also be considered, as ATM inhibition sensitizes cells to genotoxic insults in general, raising the concern of normal tissue toxicity [13]. We show that MLK4 acts as a novel, druggable regulator of DDR. Notably, MLK4 loss or inhibition does not potentiate cytotoxic effects of chemotherapy against normal breast epithelial cells in vitro, indicating that the further development of MLK4 inhibitors is warranted and may prove beneficial in cancer therapy. Moreover, our results suggest that MLK4 loss impairs NHEJ-mediated DSBs repair, and therefore may cause synthetic lethality in HR-deficient tumors due to the inhibition of the remaining DSBs repair pathway. This can be potentially relevant in the context of TNBC therapy, as this subtype of breast cancer is characterized by a relatively high frequency of HR-defects [50, 51].

Numerous studies have linked increased NF-κB activation and autocrine/paracrine signaling with breast cancer chemoresistance [12, 42, 43, 52, 53]. ATM plays a critical role in the induction of NF-κB transcription in response to genotoxic stress, primarily via an established nuclear-to-cytosolic signaling pathway involving NEMO, the regulatory subunit of the IKK complex [39, 54, 55]. Following MLK4 silencing, we observed lower levels of phosphorylated ATM and NEMO, impaired nuclear translocation of NF-κB, and decreased expression of pro-survival NF-κB target genes upon treatment with chemotherapy. These observations suggest that MLK4 activates NF-κB transcription indirectly via regulation of ATM-NEMO-IKK pathway upon DNA damage (Fig. 7B). Nevertheless, MLK4 also regulates NF-κB signaling by direct upstream activation of IKK, independently of DNA damage, which has been previously described in glioma and breast cancer [22, 23]. Thus, it is likely that MLK4 affects NF-κB transcriptional activity via several mechanisms in a context-dependent manner.

In summary, we demonstrate for the first time that MLK4 kinase confers chemoresistance in TNBC. We present evidence that MLK4 is involved in regulating DNA damage response signaling and contributes to chemotherapy-induced NF-κB activation, which facilitates the survival of TNBC cells. Our results indicate that MLK4 targeting can be used as a novel strategy in the treatment of chemoresistant tumors.

## Supplementary information

Supplementary Information File

Table S4

## Data Availability

The authors declare that all data supporting the findings of this study are available within the article and Supplementary File. mRNA-seq data have been deposited at GEO DataSets (GSE174692). Additional data or reagents are available from the corresponding author upon reasonable request.
