# Supplementary material for: MLK4 regulates DNA damage response and promotes triple-negative breast cancer chemoresistance
Source: Cell Death Dis. 2021 Nov 27;12(12):1111. doi: 10.1038/s41419-021-04405-0 (PMC8627512; doi:10.1038/s41419-021-04405-0)
Supplement: Supplementary file 1 — Supplementary Information File [file 41419_2021_4405_MOESM1_ESM.docx]

**Supplementary Information**

**MLK4 regulates DNA damage response and promotes triple-negative breast cancer chemoresistance**

Dawid Mehlich, Michał Łomiak, Aleksandra Sobiborowicz, Alicja Mazan, Dagmara Dymerska, Łukasz M. Szewczyk, Anna Mehlich, Agnieszka Borowiec, Monika K. Prełowska, Adam Gorczynski, Paweł Jabłoński, Ewa Iżycka-Świeszewska, Dominika Nowis, Anna A. Marusiak

**Content:**

1. **Supplementary Methods**
2. **Supplementary Figure Legends and Figures**
3. **Supplementary Tables**
4. **Supplementary References**

**Supplementary Methods**

**Generation of doxycycline-inducible cell lines**

Parental HCC1806 and SUM149PT were used to generate cells with doxycycline-inducible knock-down of MLK4, as described previously (1). To generate lentivral stock, HEK293T cells were transfected with TRIPZ shRNA_2 and shRNA_6 targeting MLK4 (Supplementary Table S1), purchased from Dharmacon. Cells were transduced with lentiviral stocks and subsequently selected with puromycin. To induce knock-down of MLK4, cells were incubated with doxycycline (Sigma) at 1 µg/ml concentration for 2-4 days.

### **CRISPR/Cas9-mediated knock-out of MLK4**

### MLK4 CRISPR/Cas9 knock-out cells were generated using InvitrogenTM TrueGuideTM Synthetic gRNA, according to the manufacturer’s instructions. Briefly, HCC1806 cells were transfected with gRNA and CAS9 protein (Thermofisher Scientific), using Lipofectamine CRISPRMAX reagent. Following transfection, the single cell clones were expanded and selected for MLK4 knock-out.

**Anchorage-dependent 2D Cas 3/7 Glo apoptotic assay**

HCC1806 and SUM149PT doxycycline-inducible cell lines were seeded into 6-well plates and incubated with 1 µg/ml doxycycline to induce MLK4 knock-down. MCF10A cells were transfected with either MLK4-targeting or control siRNA. Subsequently, cells were collected and seeded on a 96-well plate (1x10^4^ cells/well) in triplicates. The following day doxorubicin at indicated concentrations was added to the wells. After 24 hours, activity of caspases 3/7 was measured using Caspase-Glo 3/7 bioluminescent assay (Promega), according to the manufacturer’s instructions. For the luminescence measurement, microplate reader Synergy II (Bio Tek) was used.

**Anchorage-independent 3D Cas 3/7 Glo apoptotic assay**

HCC1806 and SUM149PT doxycycline-inducible cell lines were seeded on 96-well ultra-low attachment plates (Corning) at a density of 5x103 cells/well. The following day doxycycline was added to the wells at concentration 1 µg/ml to induce MLK4 knock-down and cells were incubated for an additional 48 hours. Subsequently, doxorubicin was added to the wells, and after 48 hours of treatment, activity of caspases 3/7 was measured using Caspase-Glo 3/7 bioluminescent assay as described above.

**Protein lysates preparation and immunoblotting**

For the whole cell lysates preparation, cells were lysed with RIPA (Sigma) lysis buffer supplemented with protease and phosphatase inhibitor tablets (Roche). For the isolation of nuclear proteins, cells were lysed using Nuclear Extraction Kit (Abcam), according to the manufacturer’s instructions. Proteins were resolved by SDS-PAGE gel electrophoresis and transferred onto nitrocellulose membranes (Bio-Rad). Membranes were blocked in 5% fat-free milk in TBST buffer and incubated overnight at 4 ˚C with indicated primary antibodies (Supplementary Table S2), and then for 1h with appropriate horseradish peroxidase–labeled secondary antibodies (Bio-Rad). The blots were developed with Clarity and Clarity Max substrates (Bio-Rad) and visualized using Amersham Imager 680. Images were analyzed using ImageJ.

**Immunofluorescent staining and confocal microscopy**

For *in vitro* experiments, HCC1806 cells were seeded onto coverslips and incubated with 1 µg/ml doxycycline to induce MLK4 knock-down. Subsequently, cells were washed with PBS and fixed with 4% PFA in PBS for 20 min. Cells were then permeabilized with 0.1% Triton X-100 in PBS for 15 min, washed and blocked with 10% FBS in PBS for 1 h. The primary yH2AX antibody (Supplementary Table S2) was added in 5% FBS in PBS and incubated in a humid chamber at 4°C overnight. Cells were washed and incubated with secondary antibody in PBS (Goat anti-Rabbit IgG, Alexa Fluor 488, Invitrogen). Next, the cells were washed, and coverslips were mounted on microscope slides using ProLong Diamond Antifade Mountant with DAPI (Thermofisher Scientific). For evaluation of xenograft tumors, tissues were dissected, fixed with 4% PFA and cryoprotected with 30% sucrose in PBS for 24 h. Next, samples were transferred to O.C.T. (Sakura Tissue-Tek) and frozen in −30 °C isopentane. Sections were obtained using a Leica CM1860 cryostat. Frozen free‐floating slices were washed with PBST. Antigen retrieval was performed using sodium citrate buffer (10 mM Sodium Citrate, 0.05% Tween 20, pH 6.0). Next, slices were blocked in 5% goat serum, incubated with primary antibodies (Supplementary Table S2) and secondary antibodies (Goat anti-Rabbit IgG DyLight 405, Invitrogen). Finally, slices were assembled using Fluromount G. All images were captured with Axio Imager Z2 LSM 700 Zeiss Confocal Microscope. Image analysis was performed using FIJI or ImageJ.

***In vitro* kinase assay**

Human glutathione S-transferase (GST)-tagged MLK4 kinase domain expressed using baculovirus expression system (Carna Biosciences) was incubated with kinase-inactive MKK7 (Carna Biosciences) in the absence or presence of inhibitor. Kinase assay was performed with 200 mM ATP at 30 ˚C for 30 min. Following addition of 4x reduced SDS sample buffer, proteins were resolved by SDS–PAGE and analyzed by western blotting.

**LC-MS/MS phosphoproteomic analysis**

Cell lysates were obtained in different experimental conditions as indicated. Lysates were digested with 8 µg of trypsin overnight at 37^o^C overnight. Next, samples were purified using SEP-PAK cartridges and subjected to phosphopeptides enrichment using TiO_2_ beads (GL Sciences). Phospho-enriched samples were analyzed by LC-MS/MS. Mass spectrometry measurements were performed at the Mass Spectrometry Laboratory at the Institute of Biochemistry and Biophysics PAS. Chromatographic separation was performed on an Evosep EV1106 analytical column. Each sample was measured in duplicate. An Evosep One nano-LC system (Evosep) was coupled to a FAIMS interface-equipped Exploris 480 mass spectrometer via an Flex ion source (Thermo Fisher Scientific). The Exploris 480 was operated in data-dependent mode with survey scans acquired at a resolution of 60,000 at m/z 200. Up to 25 of the most abundant isotope patterns with charges 2-6 from the survey scan were selected with an isolation window of 1.6 m/z and fragmented by higher-energy collision dissociation (HCD) with normalized collision energies of 30%, while the dynamic exclusion was set to 20 s. The maximum ion injection times for the survey scan and the MS/MS scans (acquired with a resolution of 30,000 at m/z 200) were set to Auto. The normalized ion target value for MS was set to 300% and for MS/MS to Standard. To increase the resolution for TMT reporter ions the TurboTMT algorithm was applied.

The data were processed with MaxQuant v. 1.6.10.43 and the peptides were identified from the MS/MS spectra searched against the UniprotKB Human Proteome using the built-in Andromeda search engine. Peptide N-terminal/Lys TMT and Cys carbamidomethylation were set as a fixed modification and Ser/Thr/Tyr phosphorylation, Met oxidation, and Asn/Gln deamidation were set as variable modifications. For in silico digests of the reference proteome, cleavages of arginine or lysine followed by any amino acid were allowed (trypsin/P), and up to two missed cleavages were allowed. The FDR was set to 0.01 for peptides, proteins, and sites. Match between runs was enabled and second peptides disabled. Other parameters were used as pre-set in the software. Corrected (based on the correction factors provided by the manufacturer for the TMT batch utilized) of phosphorylation sites’ TMT intensities were loaded into Perseus v. 1.6.10.0. Standard filtering steps were applied to clean up the dataset: reverse (matched to decoy database) and potential contaminants (from a list of commonly occurring contaminants included in MaxQuant) were removed. TMT intensities for the phosphorylation sites were log2 transformed, modification site table expanded, and sites with less than 1 valid value filtered off. Gaussian distribution of log2 transformed TMT intensities were confirmed by histogram analysis. Normalization was then applied by subtracting median values in each TMT channel. Student t-testing (permutation-based FDR with 250 randomizations = 0.05, S0 = 0.1, 2-sided, paired) was performed on the dataset to return sites in which phosphorylation levels were statistically significantly changed in response to MLK4 KD and/or drug treatment.

**RNA isolation and qRT-PCR**

RNA was isolated using the RNAeasy Mini Kit (Qiagen) according to the manufacturer’s instructions. RNA concentration was determined by the absorbance measurements using DS-11 Series Spectrophotometer/Fluorometer (DeNovix). cDNA was synthesized from 400 ng of total RNA using AMV Reverse Transcriptase (EurX). Quantitative real-time PCR was carried out using SYBR Green I (Bio-Rad) as a fluorophore. All qPCR reactions were performed on 96-well plates (Axygen), using Light Cycler 480 instrument (Roche). Samples were assayed in duplicates, and the obtained CT values were used to calculate relative gene expression using the 2^−ΔCT^ method. For each gene, the expression was normalized to the expression of B2M and actin housekeeping genes. The primers used in this study are listed in Supplementary Table S3.

**RNA-seq data processing**

For data analysis, raw sequences were trimmed according to quality using Trimmomatic using default parameters, except MINLEN, which was set to 50. Trimmed sequences were mapped to human reference genome provided by ENSEMBL, (version grch38_snp_tran) using Hisat2 default parameters. Optical duplicates were removed using MarkDuplicates tool from GATK with default parameters except OPTICAL_DUPLICATE_PIXEL_DISTANCE set to 12000. Mapped reads were associated with transcripts from GRCh38 database with default parameters except –stranded set to “reverse”. Differentially expressed genes were selected using DESeq2 package. Fold change was corrected using apeglm. Overrepresentation of Gene Ontology (The Gene Ontology Consortium 2019) terms and Kyoto Encyclopedia of Genes and Genomes (KEGG) categories among the top 5% of genes (according to p-value) was assessed with clusterprofiler package. Significance of association between the top 5% of genes and KEGG pathways was further tested with pathway regulation score. GSEA analysis was performed with phenoTest package. All mRNA-seq data have been deposited at GEO DataSets (GSE174692).

**Supplementary Figure Legends and Figures**

**Figure S1. A-C,** Following the transfection with MLK4-targeting or control siRNA, cells were treated with doxorubicin and etoposide for 48 h. Next, cells were stained with AnnexinV-FITC, and analyzed by flow cytometry. The representative histograms showing AnnexinV–FITC staining are shown for HCC1806, SUM149PT and MDA-MD-436 cell lines.


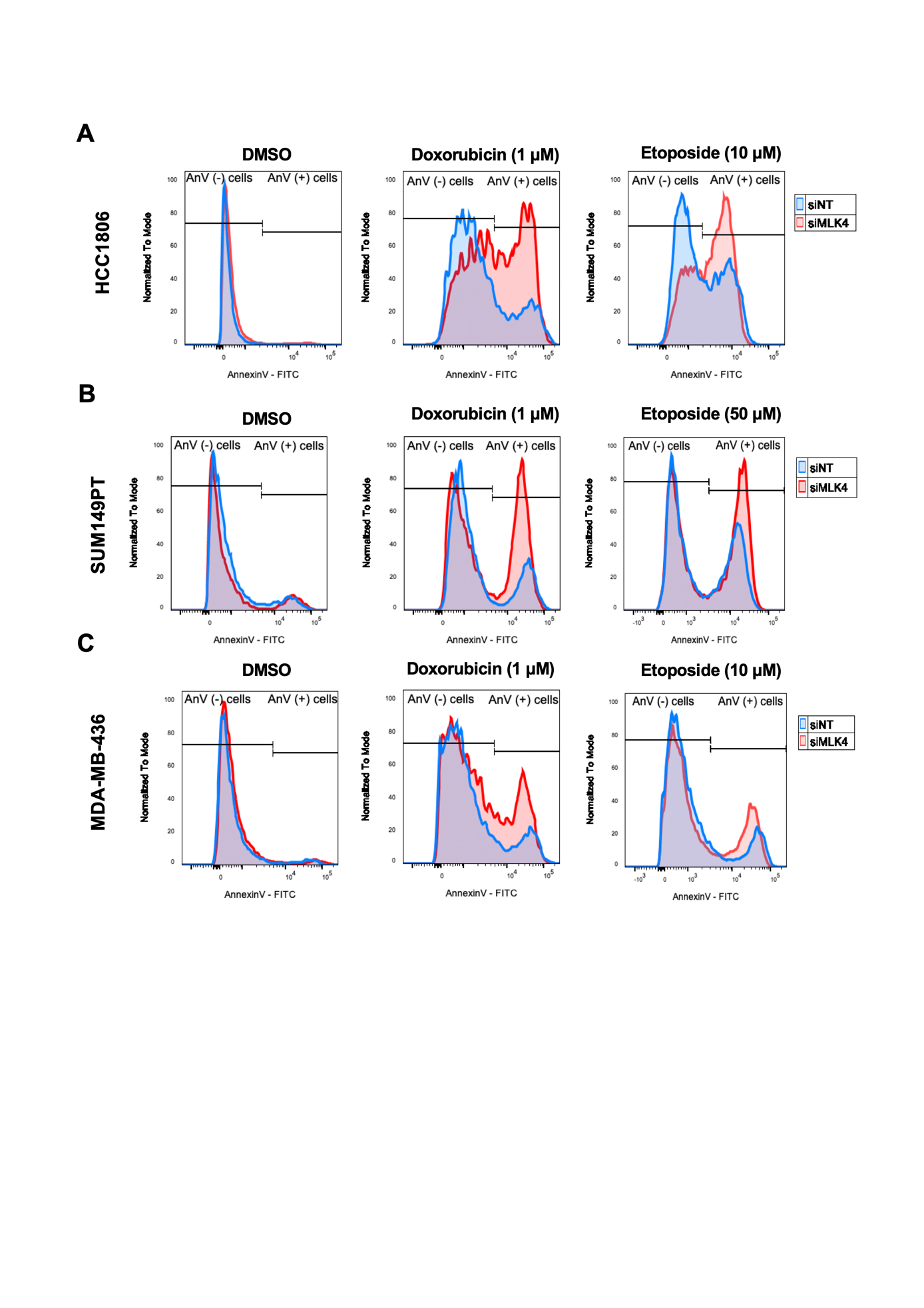


**Figure S2. A,** HCC1806 were transfected with MLK4-targeting siRNA smart-pool (siMLK4-sp) or with four individual MLK4-targeting siRNAs (siMLK4 1-4). The knock-down efficacy was assessed by immunoblotting. Cells transfected with non-targeting control siRNA (siNT) were used as a control. **B,** After the treatment, cell viability was assessed by crystal violet staining and quantified by absorbance measurements. Error bars indicate ±SEM from two independent experiments performed in triplicates (N=2, n=6). Significance was calculated using one-way ANOVA followed by Tukey multiple comparisons test, *p<0.05, **p<0.01.

**
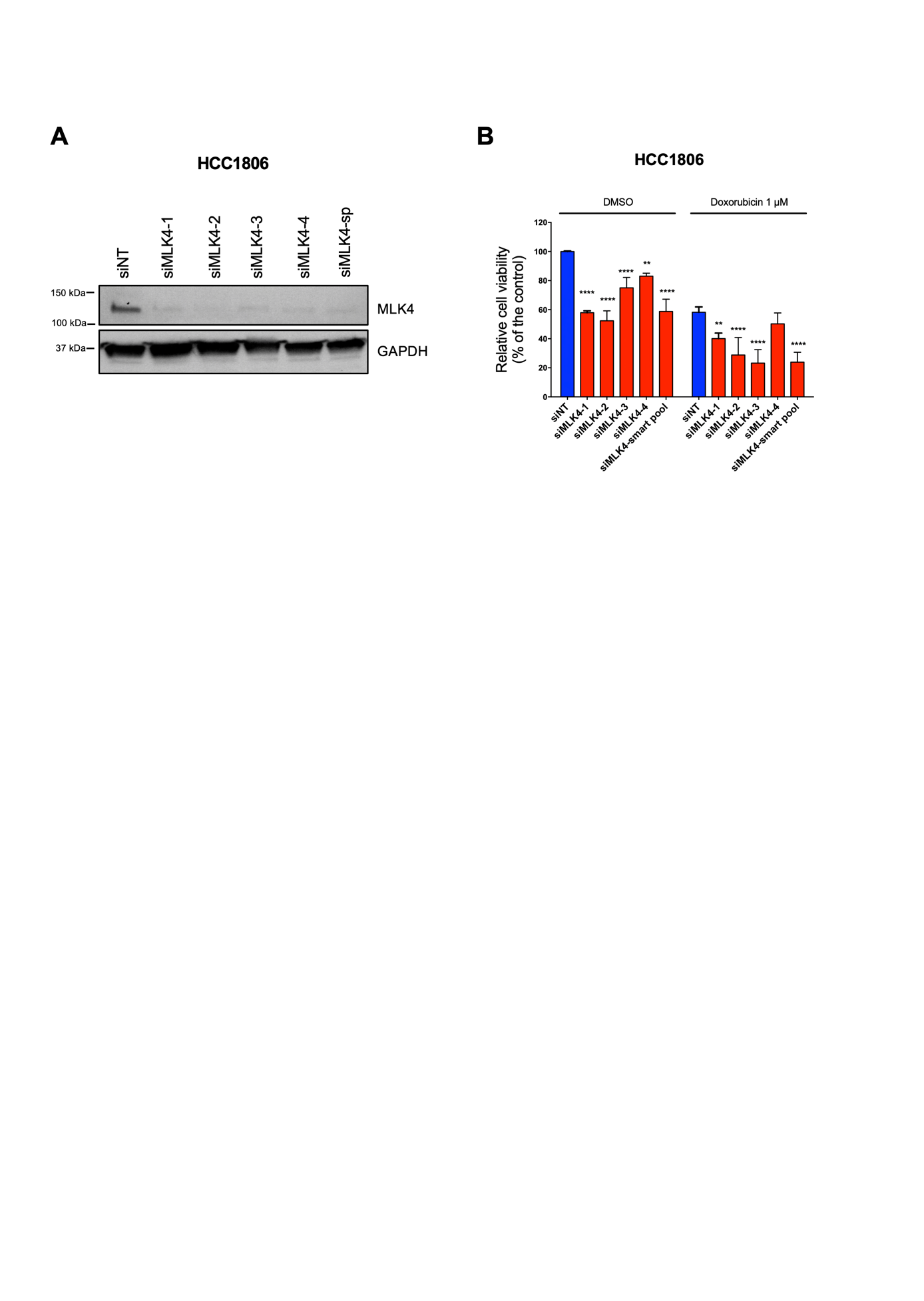
**

**Figure S3. A-D,** HCC1806 and SUM149PT parental cell lines were incubated with doxycycline and subsequently cells were treated with doxorubicin (A-B) or etoposide (C-D) for 48 h. Following treatment, cells viability was assessed by crystal violet staining and quantified by absorbance measurements. Error bars indicate ±SEM from three independent experiments, performed in triplicates. Significance was calculated using one-way ANOVA followed by Tukey multiple comparisons test, ns: not significant. **E-F,** HCC1806 and SUM149PT parental cell lines were incubated with doxycycline and subsequently treated with doxorubicin at increasing concentrations for 24 h. The activity of caspases 3/7 was measured using bioluminescence assay. Error bars indicate ±SEM from three independent experiments, performed in triplicates. Significance was calculated using an unpaired two-tailed *t*-test.

**
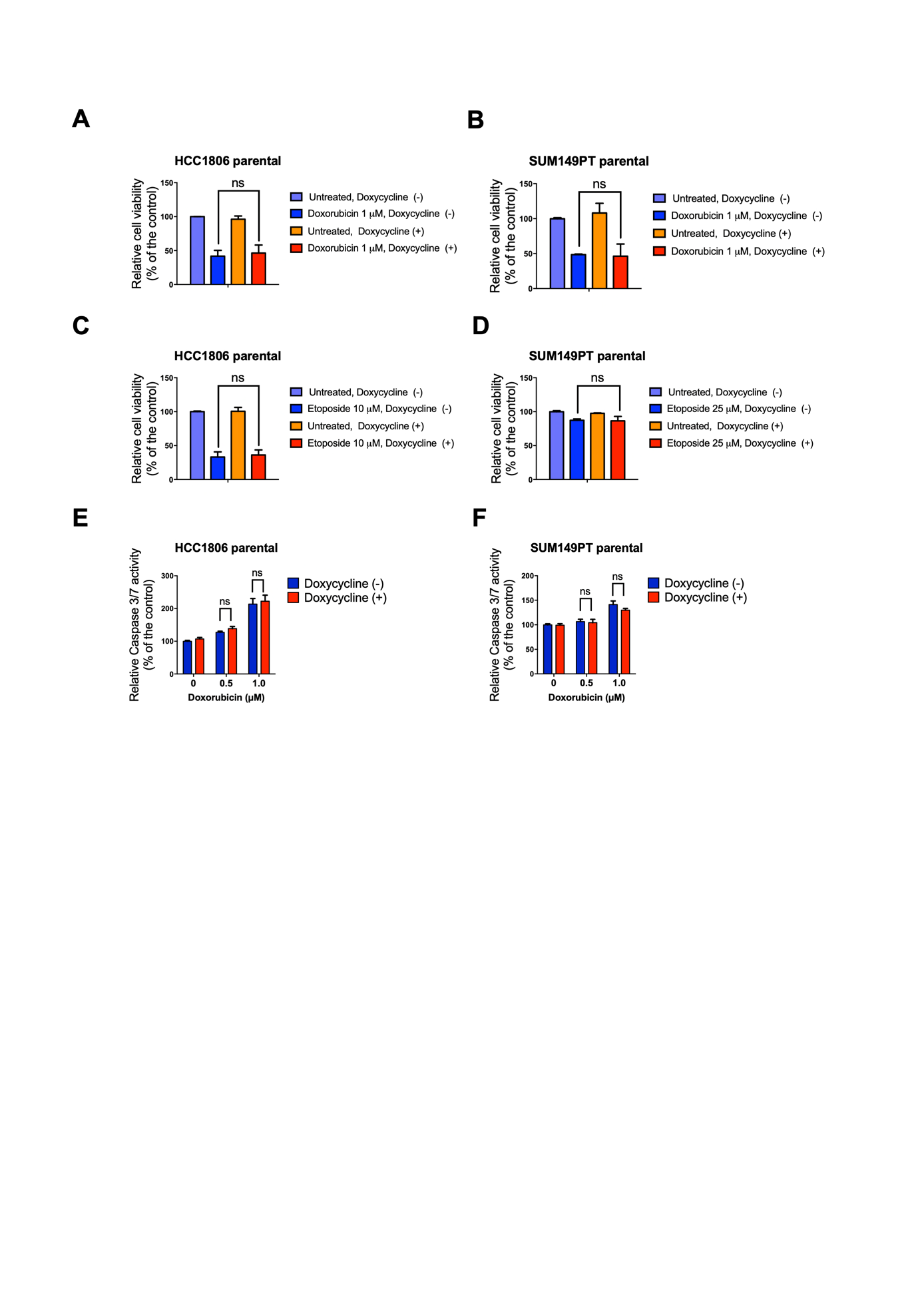
**

**Figure S4. A,** Cell lines with doxycycline-inducible MLK4 overexpression were generated from HCC1806 parental cells using lentiviral vectors. MLK4 overexpression was confirmed by immunoblotting. **B** Cells were incubated with doxycycline to induce MLK4 expression and subsequently were treated with doxorubicin at indicated concentrations. Following treatment, cell viability was assessed by crystal violet staining and quantified by absorbance measurements. Error bars indicate ±SEM from three independent experiments (n=3). Significance was calculated using an unpaired two-tailed *t*-test, **p<0.01.

**
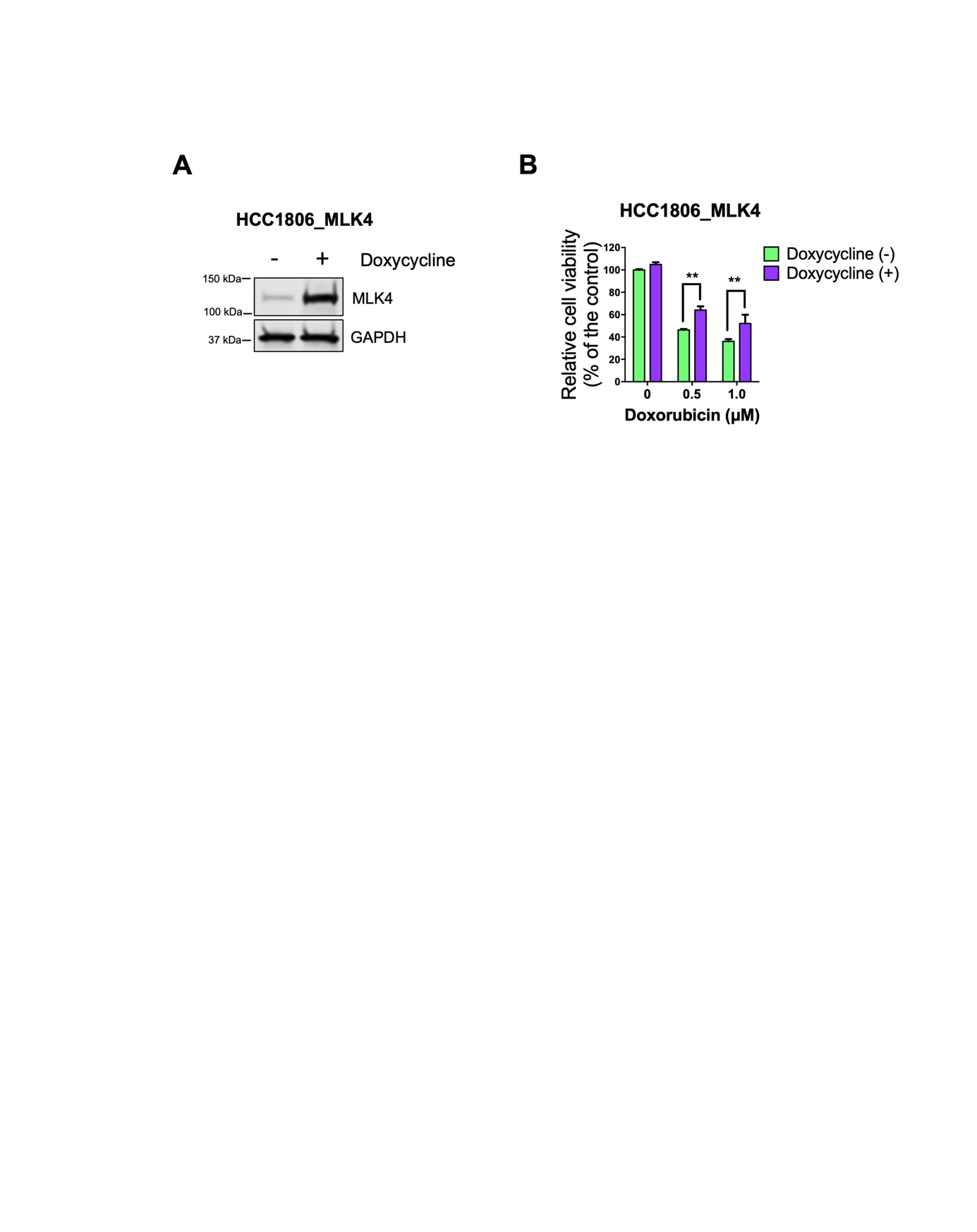
**

**Figure S5. A,** MCF10A cells were transfected using siRNA against MLK4 (siMLK4) or non-targeting siRNA control (siNT). MLK4 expression was analyzed by immunoblotting. Lysates from SUM149PT cells were run in parallel as a reference with high MLK4 expression. **B,** Following the transfection with either MLK4-targeting (siMLK4) or non-targeting control siRNA (siNT), cells were incubated with doxorubicin and etoposide at indicated concentrations for 48 h. After the treatment, cell viability was assessed by crystal violet staining and quantified by absorbance measurements. Error bars indicate ±SEM from three independent experiments (n=3). Significance was calculated using an unpaired two-tailed *t*-test. **C,** MLK4-depleted and control cells were incubated with doxorubicin and etoposide at indicated concentrations for 48 h. Next, cells were stained with AnnexinV-FITC, and analyzed by flow cytometry. Error bars indicate ±SEM from three or four independent experiments, performed in triplicates. Significance was calculated using an unpaired two-tailed *t*-test. **D,** MLK4-silenced and control cells were treated with doxorubicin at increasing concentrations and the activity of caspases 3/7 was measured using bioluminescence assay. Error bars indicate ±SEM from three independent experiments, performed in duplicates (n=6). Significance was calculated using an unpaired two-tailed *t*-test. **E,** MCF10A cells were incubated with CEP-5214 (250 nM) or DMSO for 72 h and doxorubicin for 48 h. After treatment, cells viability was assessed by crystal violet staining and quantified by absorbance measurements. Error bars indicate ±SEM from three independent experiments. Significance was calculated using one-way ANOVA followed by Tukey multiple comparisons. **F,** MCF10A cells were incubated with CEP-5214 (250 nM) or DMSO for 72 h and doxorubicin for 48 h, then stained with AnnexinV-FITC, and measured by flow cytometry. Error bars indicate ±SEM from two independent experiments, performed in triplicates (n=6). Significance was calculated using one-way ANOVA followed by Tukey multiple comparisons. For the statistical comparisons - ns: not significant.

**
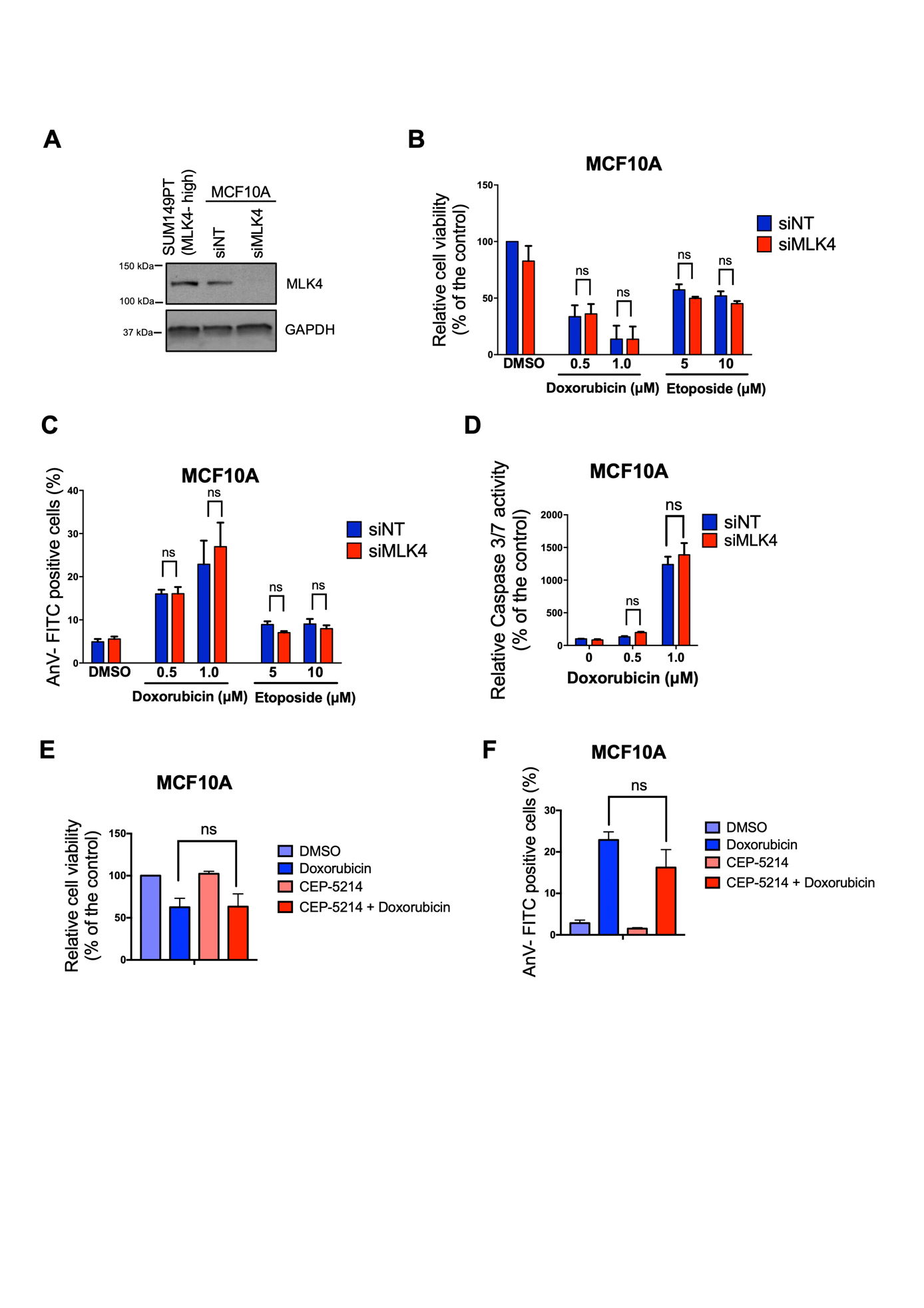
**

**Figure S6. A-B,** MLK4 knock-down was induced in HCC1806_sh6 and SUM149PT_sh2 cell lines by doxycycline, and cells were subsequently treated with neocarzinostatin at concentration of 100 ng/ml for indicated time. After the treatment, whole cell lysates were collected and analyzed by immunoblotting.


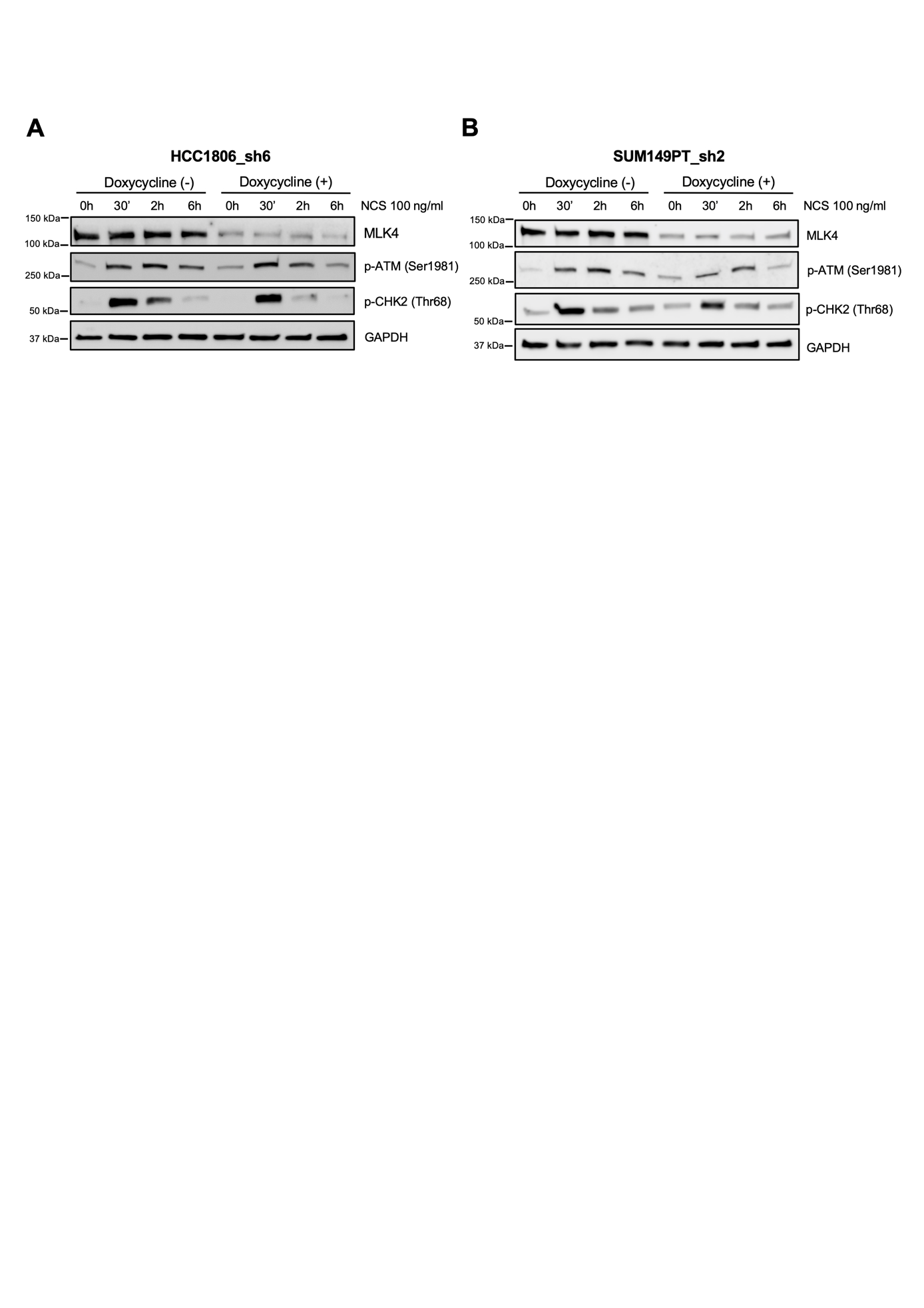


**Figure S7. A,** MLK4 knock-down in U2OS cells transfected with MLK4-targeting siRNA (siMLK4). Cells transfected with non-targeting siRNA (siNT) were used as a control. **B,** U2OS-pDR-GFP and U2OS-pimEJ5-GFP cell lines were generated from parental cells lines by transfection of the appropriate vectors and subsequent selection with puromycin for over 14 days. The cells stably expressing reporter vectors were transfected with MLK4-targeting siRNA (siMLK4) or control non-targeting siRNA (siNT) along with pSCE-CbaI endonuclease expressing vector. After 72 h, the activity of homologous recombination (pDR-GFP vector expressing cells) and non-homologous end joining (pimEJ5-GFP vector expressing cells) DNA repair pathways was assessed by measuring the percentage of GFP-positive cells using flow cytometry. Error bars indicate ±SEM from three independent experiments performed in triplicates (n=9). Significance was calculated using an unpaired two-tailed *t*-test ****p<0.0001.

**
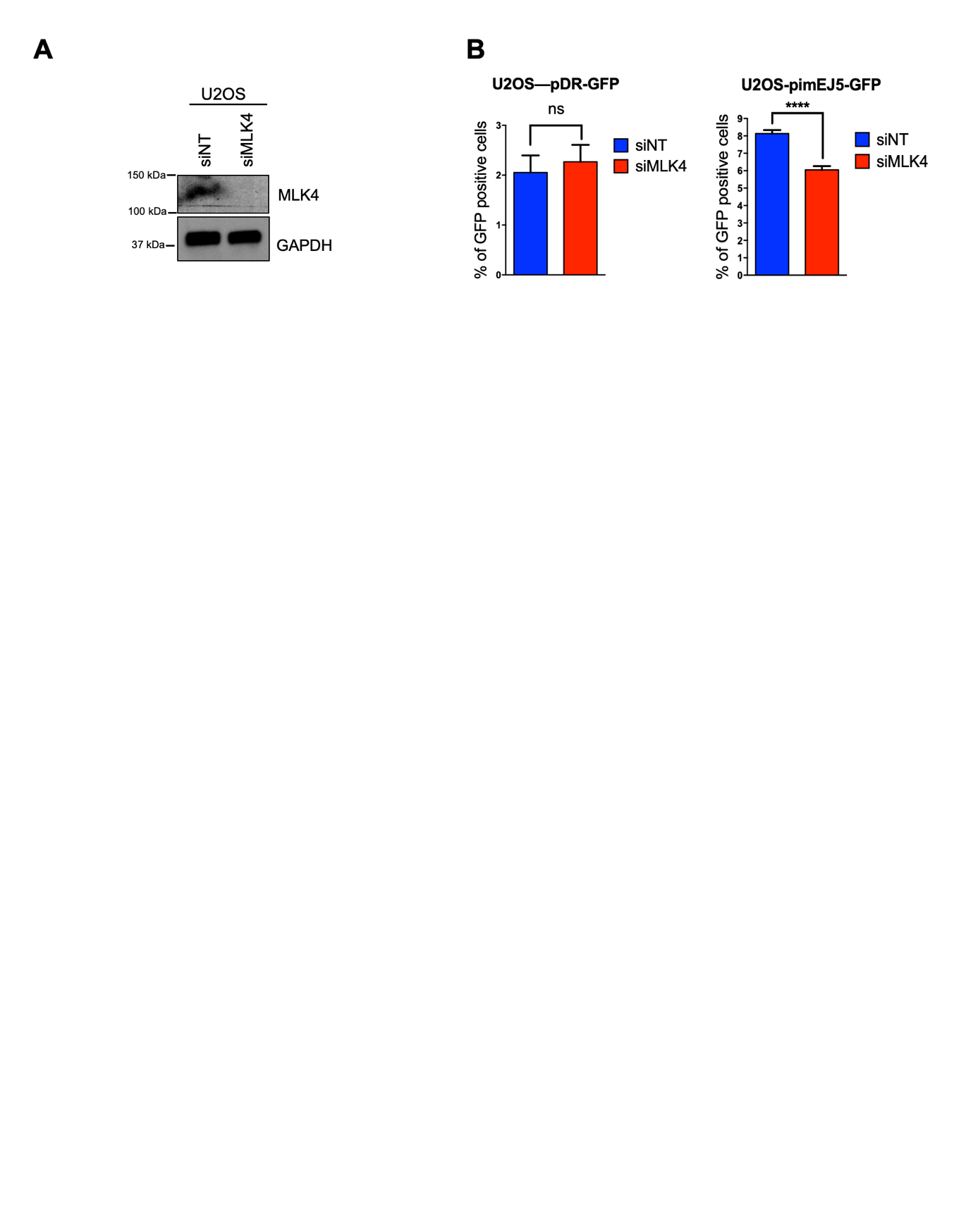
**

**Figure S8. A,** Principal component analysis plot obtained after RNA-seq of HCC1806 transfected with MLK4-targeting siRNA or control siRNA, either treated with DMSO or doxorubicin for 24 h. **B,** Venn diagram comparing numbers of transcripts significantly up (UP) or down-regulated (DW) by doxorubicin treatment in each dataset. In control cells, 1958 transcripts were upregulated and 2159 down regulated by doxorubicin. Among these transcripts, 1541 were found to be commonly upregulated in both groups, while 558 and 416 transcripts were uniquely upregulated in MLK4-depleted and control cells, respectively.

**
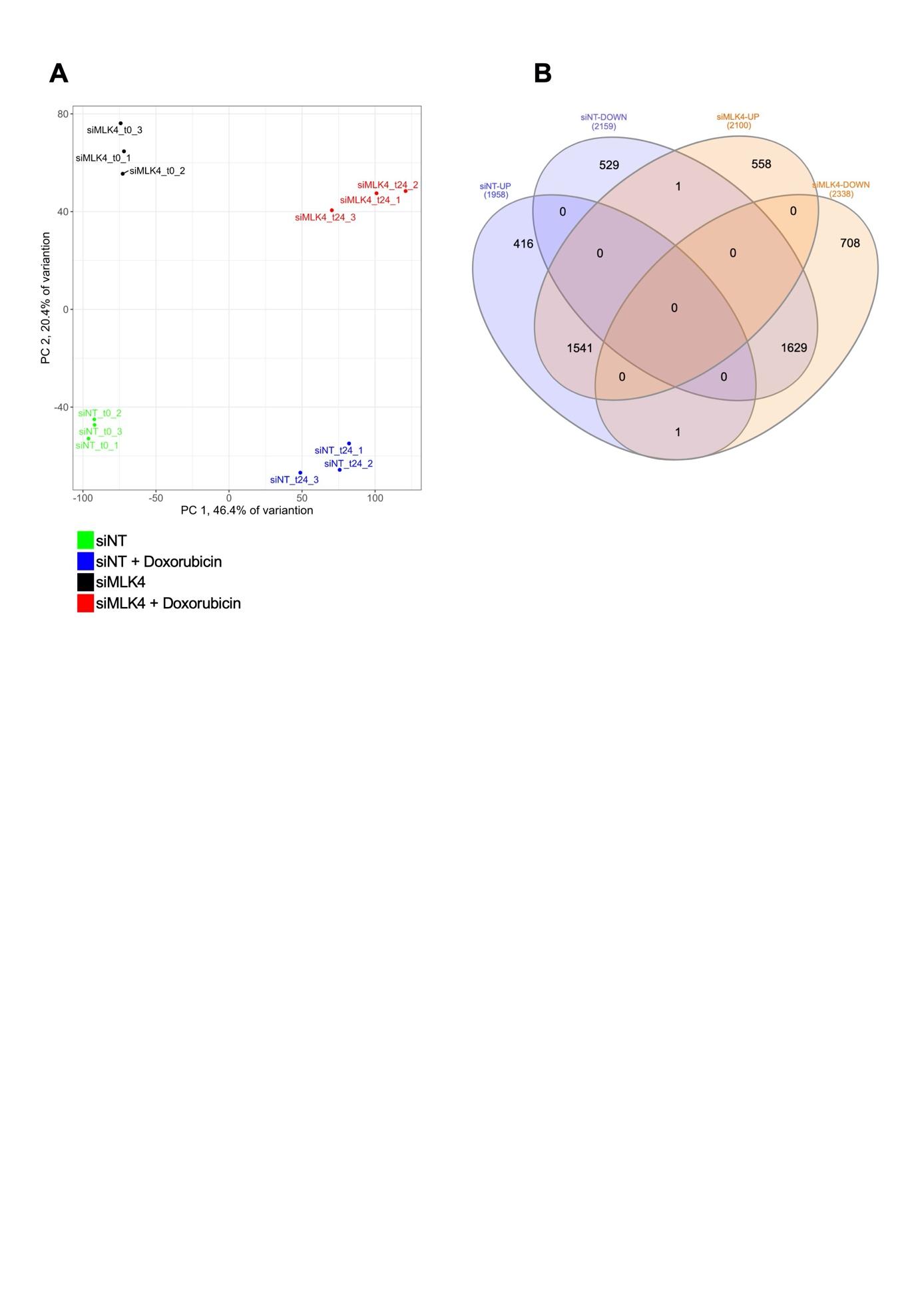
**

**Figure S9. A-B,** HCC1806 and SUM149PT cells were treated with doxorubicin, ATM inhibitor: KU-60019 or combination of both for 48h. After the treatment, cell viability was assessed by crystal violet staining and quantified by absorbance measurements. Error bars indicate ±SEM from two independent experiments, performed in triplicates. Significance was calculated using one-way ANOVA followed by Tukey multiple comparisons, ****p<0.0001. **C,** MLK4 knock-down was induced by doxycycline in HCC1806_sh6 cell line, and subsequently cells were treated with doxorubicin for indicated time. Following the treatment, whole cell lysates were collected and analyzed by immunoblotting. **D,** Nuclear extracts were isolated from MLK4-depleted and control HCC1806_sh6 cells and analyzed by immunoblotting.

**
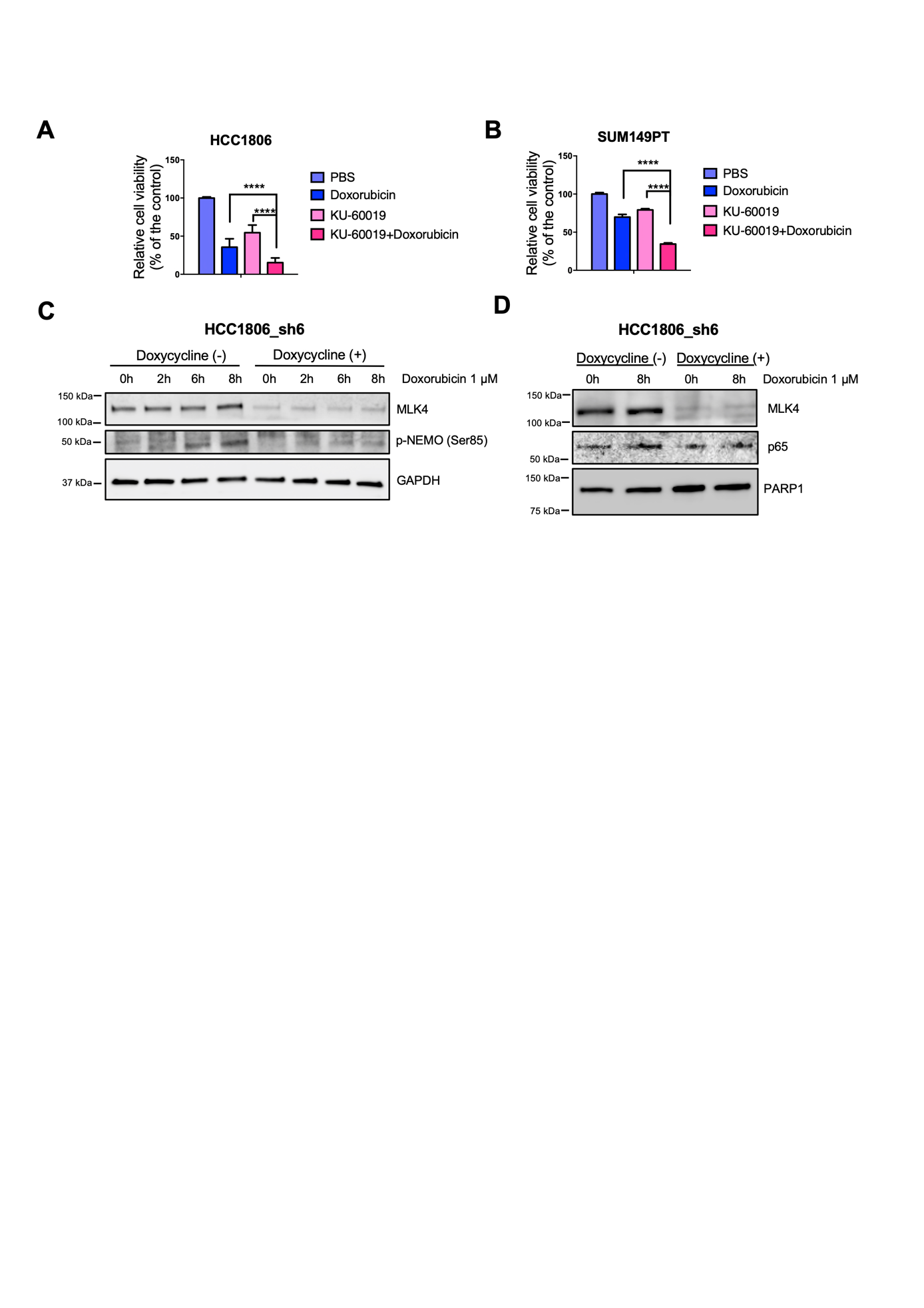
**

**Supplementary Tables**

**Table S1. List of siRNA and shRNA sequences.**

| **siRNA** | **Sequence** |
| --- | --- |
| siMLK4 #1 | GGAAAGAUGCUCAGAGAGAUU |
| siMLK4 #2 | AGGAGAAGCCCAAGGUAAAUU |
| siMLK4 #3 | AGAAGAAACGAGAGGGAAUUU |
| siMLK4 #4 | AGAACAGAUUGCAAAGAAAUU |
| **shRNA** | **Sequence** |
| sh_2 | ATCAGAATGTTAAGTTCCC |
| sh_6 | TCTTGATACACTACAATCA |

**Table S2. List of antibodies used in this study.**

| **Antibody** | **Species** | **Source** | **Catalog no.** | **Dilution** |
| --- | --- | --- | --- | --- |
| p-NEMO | Rabbit | Abcam | ab63551-100 | 1:1000 |
| MLK4 | Rabbit | Bethyl | A302-610A | 1:1000 |
| ATM | Rabbit | Bethyl | A300-299A | 1:100 |
| GAPDH | Rabbit | Cell Signaling Tech. | 2118 | 1:1000 |
| PARP | Rabbit | Cell Signaling Tech. | 9532 | 1:1000 |
| Cl. CASP3 | Rabbit | Cell Signaling Tech. | 96645 | 1:500 |
| γH2AX | Rabbit | Cell Signaling Tech. | 9718 | 1:1000 |
| p-ATM (Ser1981) | Rabbit | Cell Signaling Tech. | 5883 | 1:1000 |
| ATM | Rabbit | Cell Signaling Tech. | 2873 | 1:1000 |
| p-CHK2 (Thr68) | Rabbit | Cell Signaling Tech. | 2197 | 1:1000 |
| p65 | Rabbit | Cell Signaling Tech. | 8242 | 1:1000 |
| p-JNK | Rabbit | Cell Signaling Tech. | 4671 | 1:1000 |
| p-ERK | Rabbit | Cell Signaling Tech. | 4377 | 1:1000 |
| p-MKK7 | Rabbit | Cell Signaling Tech. | 4171 | 1:1000 |
| MKK7 | Rabbit | Cell Signaling Tech. | 4172 | 1:1000 |
| GST | Rabbit | Cell Signaling Tech. | 2624 | 1:1000 |
| Flag | Mouse | Sigma | F3165 | 1:1000 |

**Table S3. List of primers used in this study.**

| **Gene** | **Forward Primer (5’-3’)** | **Reverse Primer (3’-5’)** |
| --- | --- | --- |
| MLK4 | CATGAGGAGGCCTTCGTG | CGCCAACCCAAAATCTGTAA |
| B2M | TGGAGGCTATCCAGCGTACT | CGGATGGATGAAACCCAGAC |
| β-actin | CATCCTCACCCTGAAGTACC | AGCCTGGATAGCAACGTACAT |
| IL-6 | GACCCAACCACAAATGCCA | GTCATGTCCTGCAGCCACTG |
| IL-8 | CTGGCCGTGGCTCTCTTG | CCTTGGCAAAACTGCACCTT |
| IL-12A | ACCACTCCCAAAACCTGC | CCAGGCAACTCCCATTAG |
| CXCL1 | AGCTTGCCTCAATCCTGCATCC | TCCTTCAGGAACAGCCACCAGT |
| CXCL6 | GGGAAGCAAGTTTGTCTGGACC | AAACTGCTCCGCTGAAGACTGG |
| TNFSF15 | AAGGACAGGAGTTTGCACCTTCA | AAGTGCTGTGTGGGAGTTTGTCT |

**Table S4. Quantitative phosphoproteomics results (as separate file).**

**Supplementary References**

1. A. A. Marusiak *et al.*, “Upregulation of MLK4 promotes migratory and invasive potential of breast cancer cells,” *Oncogene*, vol. 38, no. 15, Art. no. 15, Apr. 2019, doi: 10.1038/s41388-018-0618-0.
